# Syntheses and crystal structures of four 4-(4-nitro­phen­yl)piperazinium salts with hydrogen succinate, 4-amino­benzoate, 2-(4-chloro­phen­yl)acetate and 2,3,4,5,6-penta­fluoro­benzoate anions

**DOI:** 10.1107/S2056989023001093

**Published:** 2023-02-09

**Authors:** Vinaya, Yeriyur B. Basavaraju, Hemmige S. Yathirajan, Sean Parkin

**Affiliations:** a Department of Studies in Chemistry, University of Mysore, Manasagangotri, Mysuru-570 006, India; b Department of Chemistry, University of Kentucky, Lexington, KY, 40506-0055, USA; University of Aberdeen, United Kingdom

**Keywords:** crystal structure, piperazinium salt, symmetrical hydrogen bond, crystal structure

## Abstract

The syntheses and crystal structures are presented for four organic salts of the 4-(4-nitro­phen­yl)piperazinium cation, namely, 4-(4-nitro­phen­yl)piperazinium hydrogen succinate, C_10_H_14_N_3_O_2_^+^·C_4_H_5_O_4_^−^ (**I**), 4-(4-nitro­phen­yl)piperazinium 4-amino­benzoate monohydrate, C_10_H_14_N_3_O_2_^+^·C_7_H_6_NO_2_^−^·H_2_O (**II**), 4-(4-nitro­phen­yl)piperazinium 2-(4-chloro­phen­yl)acetate, C_10_H_14_N_3_O_2_^+^·C_8_H_6_ClO_2_^−^ (**III**) and 4-(4-nitro­phen­yl)piperazinium 2,3,4,5,6-penta­fluoro­benzoate, C_10_H_14_N_3_O_2_^+^·C_7_F_5_O_2_^−^ (**IV**). The salts form from mixtures of *N*-(4-nitro­phen­yl)piperazine and the corresponding acid [succinic acid (**I**), 4-amino­benzoic acid (**II**), 2-(4-chloro­phen­yl)acetic acid (**III**) and 2,3,4,5,6-penta­fluoro­benzoic acid (**IV**)] in mixed solvents of methanol and ethyl acetate. Salts **I**, **III**, and **IV** are anhydrous, whereas **II** is a monohydrate. In each structure, the overall conformation of the cation is determined by the disposition of the exocyclic N—C bond of the piperazine ring (either axial or equatorial) and twists about the N—C bond between the piperazine ring and its attached 4-nitro­phenyl ring. The packing motifs in each structure are quite different, though all are dominated by strong N—H⋯O hydrogen bonds, which are augmented in **I** and **II** by O—H⋯O hydrogen bonds, and in **III** by a π–π stacking inter­action between inversion-related 4-nitro­phenyl groups.

## Abstract 2

The syntheses and low-temperature crystal structures of four organic salts of 4-(4-nitro­phen­yl)piperazine are presented.

## 1. Chemical context

4-Nitro­phenyl­piperazinium chloride monohydrate has been used as an inter­mediate in the synthesis of anti­cancer drugs, transcriptase inhibitors and anti­fungal reagents (Berkheij *et al.*, 2005 ▸; Chaudhary *et al.*, 2006 ▸; Kharb *et al.*, 2012 ▸; Upadhayaya *et al.*, 2004 ▸). It is also an important reagent for potassium channel openers, which show significant biomolecular current-voltage rectification characteristics (Lu, 2007 ▸). The design, synthesis and biological profiling of aryl­piperazine-based scaffolds for the management of androgen-sensitive prostatic disorders was described by Gupta *et al.* (2016 ▸). 4-Nitro­phenyl­piperazine was the starting material in the synthesis and biological evaluation of new piperazine-containing hydrazone derivatives (Kaya *et al.*, 2016 ▸). A review on the piperazine skeleton in the structural modification of natural products was recently published by Zhang *et al.* (2021 ▸).

As part of our studies in this area, this paper describes the crystal structures of four salts of 4-nitro­phenyl­piperazine with organic acids, *viz*, 4-(4-nitro­phen­yl)piperazinium hydrogen succinate, C_10_H_14_N_3_O_2_^+^·C_4_H_5_O_4_^−^ (**I**), 4-(4-nitro­phen­yl)pip­er­azinium 4-amino­benzoate monohydrate, C_10_H_14_N_3_O_2_^+^·C_7_H_6_NO_2_^−^·H_2_O (**II**), 4-(4-nitro­phen­yl)piperazinium 2-(4-chloro­phen­yl)acetate, C_10_H_14_N_3_O_2_^+^·C_8_H_6_ClO_2_^−^ (**III**) and 4-(4-nitro­phen­yl)piperazinium 2,3,4,5,6-penta­fluoro­benzoate, C_10_H_14_N_3_O_2_^+^·C_7_F_5_O_2_^−^ (**IV**). 
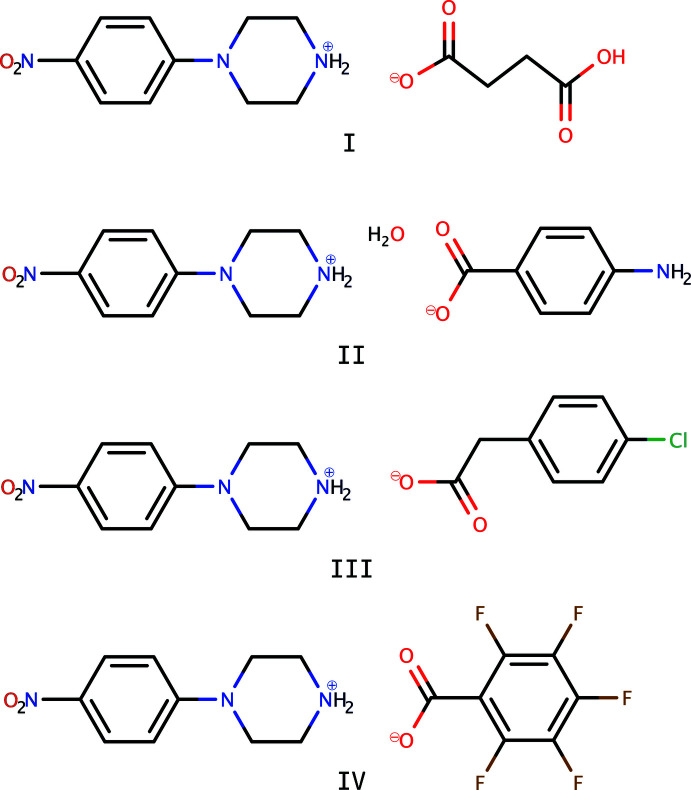


## 2. Structural commentary

**Figure 1:**
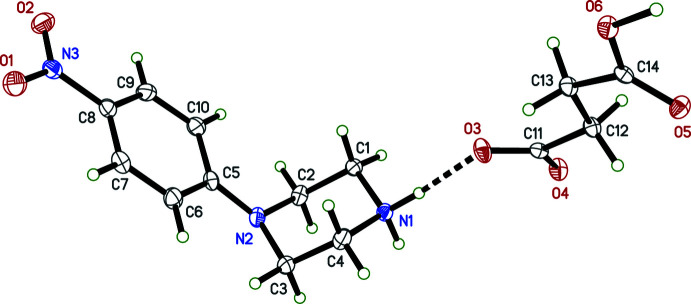
The mol­ecular structure (50% displacement ellipsoids) of **I**. Hydrogen atoms are shown as arbitrary circles. The dashed line indicates a hydrogen bond.

The overall conformations of the 4-nitro­phenyl­piperazinium cations in **I**–**IV** are determined by the N2—C5 bonds, which link the 4-nitro­phenyl and piperazinium rings (Figs. 1 ▸–4 ▸ ▸ ▸). Within each structure, atom N2 is non-planar, the sums of bond angles being 352.73 (16)° (**I**), 344.91 (12)° (**II**), 348.75 (15)° (**III**), and 348.85 (17)° (**IV**), so the connection of the exocyclic carbon atom is either equatorial (**II**, **III**) or axial (**I**, **IV**). The relative twist about these N2—C5 bonds, *e.g.* qu­anti­fied by the C2—N2—C5—C6 torsional angles [–168.06 (10)° for **I**, 149.97 (9)° for **II**, 167.32 (10)° for **III**, and −170.03 (10)° for **IV**] determine the overall cation shape. In each case, the 4-nitro group is essentially coplanar with its attached phenyl ring.

The succinate anion in **I** has minor twists about its three C—C bonds [torsion angles 165.46 (9), 166.06 (8), and 169.97 (9)° for O4—C11—C12—C13, C11—C12—C13—C14, and C12—C13—C14—O6, respectively], which leads to a dihedral angle of 34.63 (9)° between its carboxyl­ate/carb­oxy­lic acid groups. The 4-amino­benzoate anion of **II** is close to planar, having a dihedral angle between the carboxyl­ate group and its benzene ring of 10.70 (7)°. The amine group at N4 is also slightly non-planar [the sum of angles about N4 is 349 (2)°]. In the 2-(4-chloro­phen­yl)acetate anion of **III**, twists about the C11—C12 and C12—C13 bonds place the carboxyl­ate group almost perpendicular [85.02 (9)°] to the benzene ring. Lastly, in the penta­fluoro­benzoate anion of **IV**, the carboxyl­ate group is 55.95 (10)° out of coplanarity with the phenyl ring.

Throughout all four structures, individual bond lengths and angles take on normal values except for an elongated O—H bond [1.17 (2) Å] in **I**, which will be described in more detail in the next section (*Supra­molecular features*).

## 3. Supra­molecular features

Hydrogen bonding plays a significant role in the packing of all four salts (see Tables 1 ▸–4 ▸ ▸ ▸). In each structure, the asymmetric units were chosen to give the shortest hydrogen bonds between the cationic NH_2_ group and the anionic carboxyl­ate groups. In **I**, **II**, and **IV**, these hydrogen bonds to the anion are equatorial relative to the piperazine ring, while that in **III** is axial. Nevertheless, in each structure, the NH_2_^+^ group acts as a hydrogen-bond donor through *both* its hydrogen atoms. In **I**, **III**, and **IV** this is to a second anion, whereas in **II** it is to the included water mol­ecule. Throughout the four structures, all conventional N—H⋯O and all but one O—H⋯O (in **I**, *vide infra*) hydrogen bonds take on normal distances and angles (Tables 1 ▸–4 ▸ ▸ ▸).

**Figure 5:**
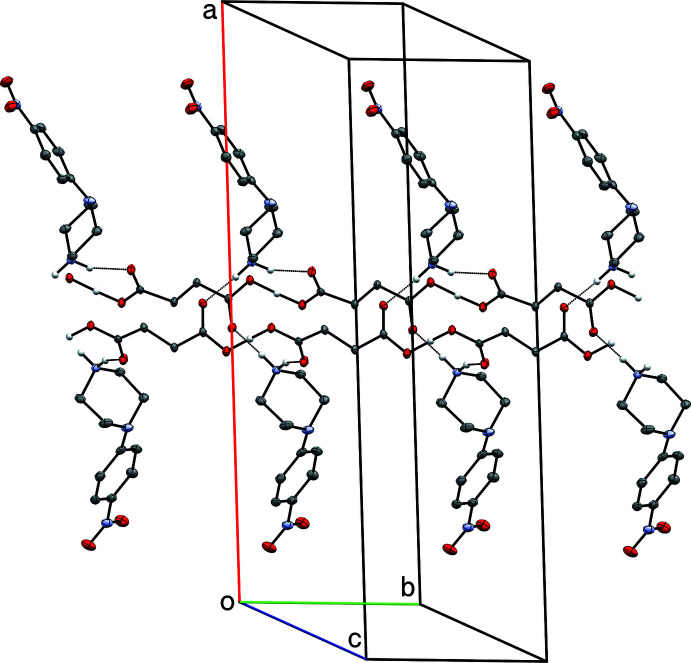
A partial packing plot of **I** showing extended double chains of O—H⋯O hydrogen-bonded (dotted lines) succinate anions, linked *via* N—H⋯O hydrogen bonds to the 4-nitro­phenyl­piperazinium cations. Hydrogen atoms not involved in hydrogen bonds are omitted.

**Figure 6:**
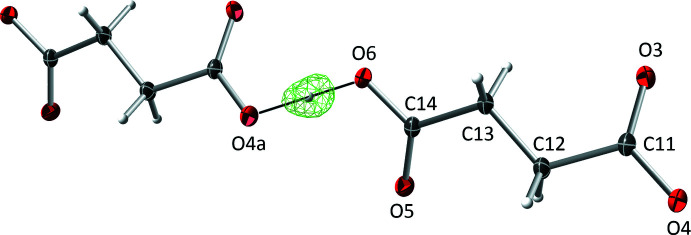
Difference-Fourier electron-density map for the region of the hydrogen atom situated close to the donor–acceptor midpoint in the short O—H⋯O hydrogen bond [thin black line, O⋯O = 2.4367 (1) Å] linking the hydrogen succinate cations into chains propagating parallel to the *b*-axis in **I**.

The structure of **I** includes an unusually short O6—H6⋯O4(*x*, *y* − 1, *z*) hydrogen bond [O⋯O = 2.4367 (10) Å], which links adjacent hydrogen-succinate anions into chains that propagate parallel to the *b*-axis direction (Fig. 5 ▸). Difference map density for this hydrogen (H6) appears roughly equidistant from both oxygen atoms (Fig. 6 ▸), and refines to give O6—H6 = 1.17 (2) Å (Table 1 ▸). For unusually strong hydrogen bonds, the migration of the hydrogen atom towards the midpoint between the donor and acceptor atoms is an expected phenomenon. In such instances, the case for positional refinement of the hydrogen atom, or even placement at the difference map peak coordinates is compelling (Fábry, 2018 ▸), and is backed by density-functional theory computational analysis (see *e.g.* Bhardwaj *et al.*, 2020 ▸). A number of weak C—H⋯O inter­actions also occur.

**Figure 7:**
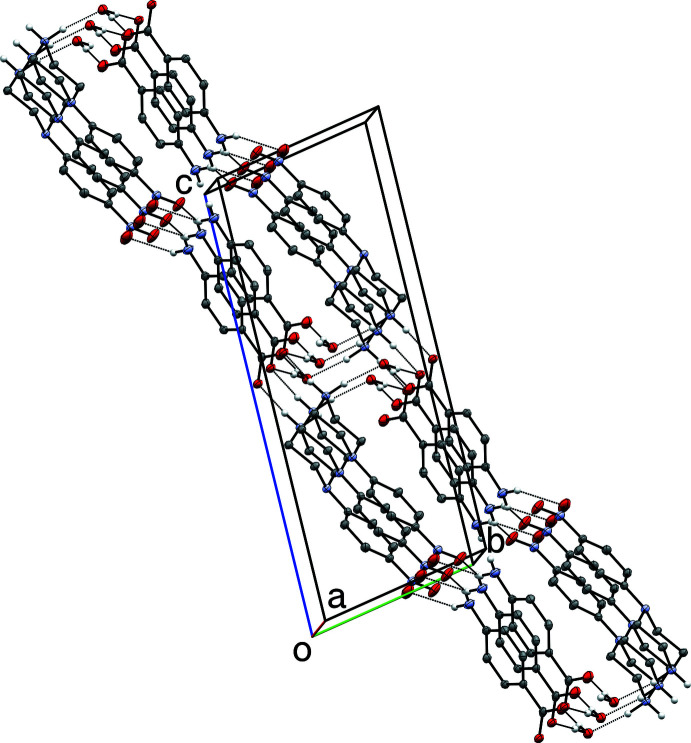
A partial packing plot of **II** showing N—H⋯O and O—H⋯O hydrogen-bonded (dotted lines) double layers that extend parallel to (011). Hydrogen atoms not involved in hydrogen bonds are omitted.

Structure **II** also includes N—H⋯O hydrogen bonds from the 4-amino group of its anion to the nitro oxygen atoms of its cation (Table 2 ▸). The cation–anion inter­actions, along with the presence of the water mol­ecule, which acts as an O—H⋯O hydrogen-bond donor to join a pair of translation-related (1 + *x*, *y*, *z*) anions and as an acceptor for an N—H⋯O hydrogen bond, generates a double-layer network lying parallel to (011) (Fig. 7 ▸). Of the four structures, **II** has the most complex hydrogen-bonding inter­actions.

**Figure 8:**
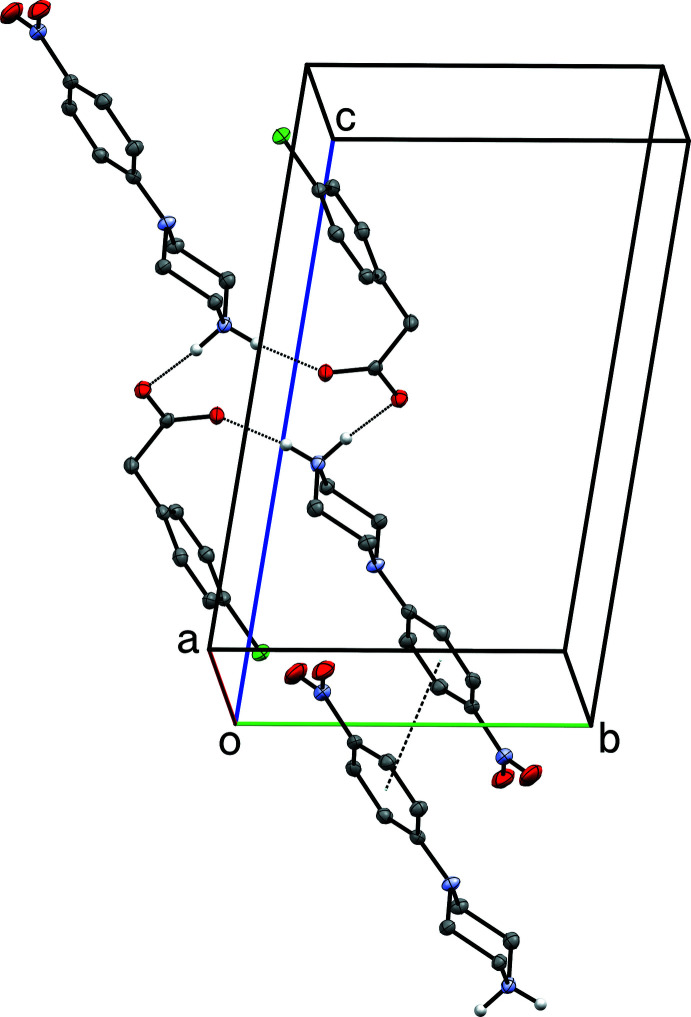
A partial packing plot of **III** showing a hydrogen-bonded (dotted lines) 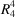 (12) ring motif and a π–π inter­action (dashed line) between inversion-related cations. Hydrogen atoms not involved in hydrogen bonds are omitted.

The primary supra­molecular inter­action in **III** joins two pairs of inversion-related ammonium cations and carboxyl­ate anions, forming an 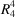 (12) ring motif (Table 3 ▸, Fig. 8 ▸). Structure **III** also includes the only π–π inter­actions of the four structures, which occurs between inversion-related (−*x*, 1 − *y*, −*z*) nitro­phenyl rings, giving an inter­planar spacing of 3.3352 (15) Å, though the offset (≃1.92 Å) is large, leading to a centroid–centroid distance of 3.8495 (15) Å (Fig. 8 ▸, dashed line).

**Figure 9:**
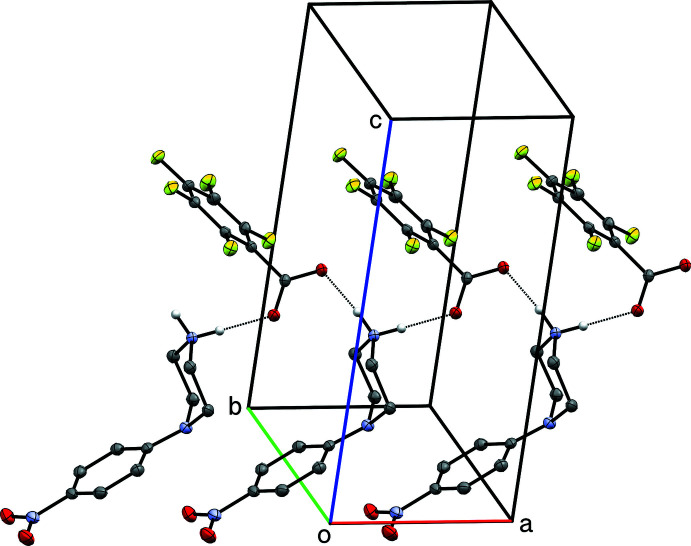
A partial packing plot of **IV**, showing chains of hydrogen-bonded (dotted lines) cations and anions that extend parallel to the *a*-axis. Hydrogen atoms not involved in hydrogen bonds are omitted.

Supra­molecular inter­actions within **IV** are the simplest of the four structures: N—H⋯O hydrogen bonds connect cations and anions into continuous chains that extend parallel to its *a*-axis. These inter­actions are qu­anti­fied in Table 4 ▸ and shown in Fig. 9 ▸.

## 4. Database survey

A search of the Cambridge Structure Database (CSD v5.43 with updates through September 2022; Groom *et al.*, 2016 ▸) for salts that include the 4-(4-nitro­phen­yl)piperazinium cation returned ten hits. Database entries with refcodes LIJNAU (Lu, 2007 ▸) and LIJNAU01 (Rehman *et al.*, 2009 ▸) are monohydrates of the chloride salt. The remaining eight structures, CSD entries NEBVOJ, NEBVUP, NEBWAW, NEBWEA, NEBWIE, NEBWOK (Mahesha *et al.*, 2022 ▸) and BEFGIG and BEFGOM (Shankara Prasad *et al.*, 2022 ▸) are all organic salts with a variety of anions, and all but NEBWOK and BEFGOM are hydrates.

Racemic perhydro­tri­phenyl­ene forms a polar inclusion compound with 1-(4-nitro­phen­yl)piperazine as a guest mol­ecule (NOVWOK; König *et al.*, 1997 ▸). The crystal structure of 4,6-di­meth­oxy­pyrimidin-2-amine-1-(4-nitro­phen­yl)piperazine (LUDMUU), was published by Wang *et al.* (2014 ▸). The synthesis and crystal structure of a Schiff base, 5-methyl-2-{[4-(4-nitro­phen­yl)piperazin-1-yl]meth­yl}phenol (WUWBIC) was published by Ayeni *et al.* (2019 ▸). NMR-based investigations by Wodtke *et al.* (2018 ▸) of acyl-functionalized piperazines concerning their conformational behavior in solution, included crystal structures of 1-(4-fluoro­benzo­yl)-4-(4-nitro­phen­yl)piperazine (BIQYIM), 1-(4-bromo­benzo­yl)-4-(4-nitro­phen­yl)piperazine (BIRHES), 1-(3-bromo­benzo­yl)-4-(4-nitro­phen­yl)piperazine (BIRHIW) and (piperazine-1,4-di­yl)bis­[(4-fluoro­phen­yl)methanone] (BIRGOB).

## 5. Synthesis, crystallization and spectroscopic details

A solution of commercially available (Sigma-Aldrich) 4-nitro­phenyl­piperazine (100 mg, 0.483 mol) in methanol (10 ml) was mixed with equimolar solutions of the appropriate acid in methanol (10 ml) and ethyl acetate (10 ml) *viz*., succinic acid (60 mg, 0.483 mol) for **I**, 4-amino­benzoic acid (69 mg, 0.483 mol) for **II**, 2-(4-chloro­phen­yl)acetic acid (85 mg, 0.483 mol) for **III**, and 2,3,4,5,6-penta­fluoro­benzoic acid (102 mg, 0.483 mol) for **IV**. The resulting solutions were stirred for 30 minutes at 333 K and allowed to stand at room temperature. X-ray quality crystals formed on slow evaporation of solutions in ethanol:aceto­nitrile (1:1) over the course of a week for all four compounds. The melting points are 398–400 K (**I**), 473–475 K (**II**), 431–435 K (**III**) and 411–415 K (**IV**).

## 6. Refinement

Crystal data, data collection, and structure refinement details are given in Table 5 ▸. All hydrogen atoms were found in difference-Fourier maps, but those bound to carbon were subsequently included in the refinement using riding models, with constrained distances set to 0.95 Å (C*sp* ^2^—H) and 0.99 Å (*R*_2_CH_2_), using *U*_iso_(H) values constrained to 1.2*U*_eq_ of the attached carbon atom. All N—H and O—H hydrogen atoms were refined freely (both coordinates and *U*_iso_).

## Supplementary Material

Crystal structure: contains datablock(s) I, II, III, IV, global. DOI: 10.1107/S2056989023001093/hb8053sup1.cif

Structure factors: contains datablock(s) I. DOI: 10.1107/S2056989023001093/hb8053Isup2.hkl

Structure factors: contains datablock(s) II. DOI: 10.1107/S2056989023001093/hb8053IIsup3.hkl

Structure factors: contains datablock(s) III. DOI: 10.1107/S2056989023001093/hb8053IIIsup4.hkl

Structure factors: contains datablock(s) IV. DOI: 10.1107/S2056989023001093/hb8053IVsup5.hkl

Supporting information file. DOI: 10.1107/S2056989023001093/hb8053Isup6.cml

Supporting information file. DOI: 10.1107/S2056989023001093/hb8053IIsup7.cml

Supporting information file. DOI: 10.1107/S2056989023001093/hb8053IIIsup8.cml

Supporting information file. DOI: 10.1107/S2056989023001093/hb8053IVsup9.cml

CCDC references: 2239906, 2239905, 2239904, 2239903

Additional supporting information:  crystallographic information; 3D view; checkCIF report

## Figures and Tables

**Figure 2:**
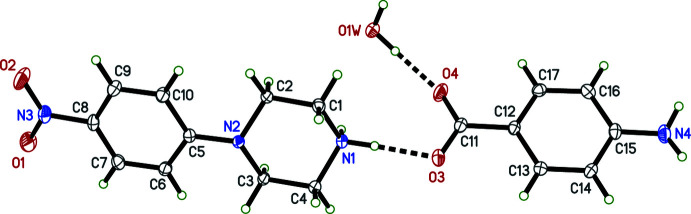
The mol­ecular structure (50% displacement ellipsoids) of **II**. Hydrogen atoms are shown as arbitrary circles. The dashed lines indicate hydrogen bonds.

**Figure 3:**
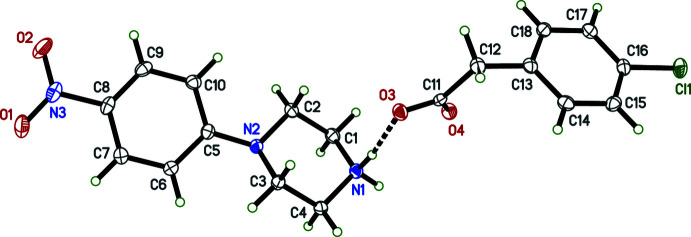
The mol­ecular structure (50% displacement ellipsoids) of **III**. Hydrogen atoms are shown as arbitrary circles. The dashed line indicates a hydrogen bond.

**Figure 4:**
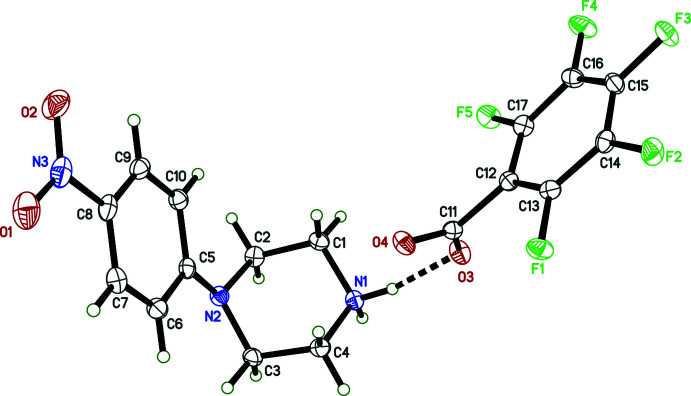
The mol­ecular structure (50% displacement ellipsoids) of **IV**. Hydrogen atoms are shown as arbitrary circles. The dashed line indicates a hydrogen bond.

**Table 1:** Hydrogen-bond geometry (Å, °) for **I** [Chem scheme1]

*D*—H⋯*A*	*D*—H	H⋯*A*	*D*⋯*A*	*D*—H⋯*A*
N1—H1*NA*⋯O3	0.934 (15)	1.793 (16)	2.7250 (12)	175.5 (13)
N1—H1*NB*⋯O5^i^	0.903 (16)	1.945 (16)	2.8127 (12)	160.5 (13)
O6—H6*O*⋯O4^ii^	1.17 (2)	1.27 (2)	2.4367 (10)	176 (2)
C1—H1*A*⋯O6^iii^	0.99	2.49	3.1374 (13)	123
C3—H3*A*⋯O5^i^	0.99	2.59	3.3238 (15)	130
C3—H3*B*⋯O2^iv^	0.99	2.51	3.4168 (15)	153
C4—H4*A*⋯O2^v^	0.99	2.55	3.5053 (15)	162
C4—H4*B*⋯O4^vi^	0.99	2.45	3.2354 (13)	136
C10—H10⋯O4^vii^	0.95	2.57	3.4146 (13)	149

Symmetry codes: (i) 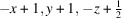 ; (ii) 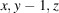 ; (iii) 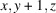 ; (iv) 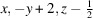 ; (v) 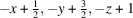 ; (vi) 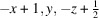 ; (vii) 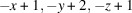 .

**Table 2:** Hydrogen-bond geometry (Å, °) for **II** [Chem scheme1]

*D*—H⋯*A*	*D*—H	H⋯*A*	*D*⋯*A*	*D*—H⋯*A*
N1—H1*NA*⋯O3	0.936 (17)	1.804 (17)	2.737 (1)	174.7 (15)
N1—H1*NB*⋯O1*W* ^i^	0.942 (15)	1.864 (15)	2.7934 (11)	168.4 (13)
N4—H4*NA*⋯O1^ii^	0.879 (18)	2.258 (18)	3.0861 (13)	156.9 (15)
N4—H4*NB*⋯O2^iii^	0.886 (18)	2.248 (17)	3.0315 (13)	147.3 (14)
O1*W*—H1*W*1⋯O3^iv^	0.877 (18)	1.890 (18)	2.7569 (11)	169.7 (16)
O1*W*—H2*W*1⋯O4	0.885 (18)	1.755 (18)	2.6388 (11)	177.2 (17)
C1—H1*C*⋯O1*W*	0.99	2.50	3.2511 (12)	132
C2—H2*B*⋯O4^i^	0.99	2.58	3.5572 (13)	170
C4—H4*A*⋯O3^v^	0.99	2.51	3.4578 (12)	161
C4—H4*B*⋯O1*W* ^vi^	0.99	2.53	3.2921 (12)	134

Symmetry codes: (i) 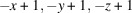 ; (ii) 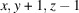 ; (iii) 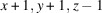 ; (iv) 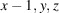 ; (v) 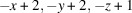 ; (vi) 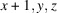 .

**Table 3:** Hydrogen-bond geometry (Å, °) for **III** [Chem scheme1]

*D*—H⋯*A*	*D*—H	H⋯*A*	*D*⋯*A*	*D*—H⋯*A*
N1—H1*NA*⋯O4^i^	0.894 (16)	1.848 (16)	2.7252 (12)	166.3 (14)
N1—H1*NB*⋯O3	0.937 (16)	1.765 (17)	2.6903 (12)	169.0 (15)
C4—H4*A*⋯O4^ii^	0.99	2.46	3.2539 (14)	137
C7—H7⋯O1^iii^	0.95	2.59	3.2256 (15)	124
C12—H12*A*⋯O3^iv^	0.99	2.49	3.4710 (14)	173
C15—H15⋯O2^v^	0.95	2.37	3.1944 (15)	146
C18—H18⋯O3^vi^	0.95	2.55	3.2644 (15)	132

Symmetry codes: (i) 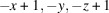 ; (ii) 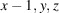 ; (iii) 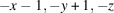 ; (iv) 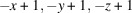 ; (v) 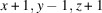 ; (vi) 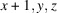 .

**Table 4:** Hydrogen-bond geometry (Å, °) for **IV** [Chem scheme1]

*D*—H⋯*A*	*D*—H	H⋯*A*	*D*⋯*A*	*D*—H⋯*A*
N1—H1*NA*⋯O3	0.929 (15)	1.754 (16)	2.6723 (13)	169.4 (14)
N1—H1*NB*⋯O4^i^	0.915 (16)	1.816 (16)	2.7310 (13)	178.8 (14)
C1—H1*C*⋯F5^ii^	0.99	2.49	3.4813 (14)	177
C1—H1*D*⋯F4^iii^	0.99	2.49	3.3996 (14)	153
C4—H4*B*⋯O3^iv^	0.99	2.56	3.3421 (15)	136
C6—H6⋯F2^v^	0.95	2.54	3.4536 (15)	161

Symmetry codes: (i) 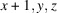 ; (ii) 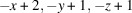 ; (iii) 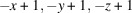 ; (iv) 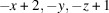 ; (v) 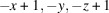 .

**Table 5:** Experimental details

	**I**	**II**	**III**	**IV**
Crystal data				
Chemical formula	C_10_H_14_N_3_O_2_^+^·C_4_H_5_O_4_^−^	C_10_H_14_N_3_O_2_^+^·C_7_H_6_NO_2_^−^·H_2_O	C_10_H_14_N_3_O_2_^+^·C_8_H_6_ClO_2_^−^	C_10_H_14_N_3_O_2_^+^·C_7_F_5_O_2_^−^
*M*_r_	325.32	362.38	377.82	419.31
Crystal system, space group	Monoclinic, *C*2/*c*	Triclinic, *P* 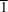	Triclinic, *P* 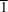	Triclinic, *P* 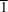
Temperature (K)	90	90	90	90
*a*, *b*, *c* (Å)	25.2747 (12), 8.0434 (4), 15.6617 (5)	6.0453 (3), 7.3930 (3), 19.1439 (6)	6.8051 (2), 9.3927 (5), 14.3869 (7)	5.9779 (3), 11.3934 (8), 12.9312 (9)
α, β, γ (°)	90, 105.384 (2), 90	79.482 (2), 89.215 (1), 83.967 (1)	83.849 (2), 81.283 (2), 72.492 (2)	75.754 (2), 81.670 (2), 87.717 (2)
*V* (Å^3^)	3069.9 (2)	836.55 (6)	865.01 (7)	844.63 (9)
*Z*	8	2	2	2
Radiation type	Mo *K*α	Mo *K*α	Mo *K*α	Mo *K*α
μ (mm^−1^)	0.11	0.11	0.25	0.15
Crystal size (mm)	0.30 × 0.22 × 0.18	0.30 × 0.26 × 0.25	0.28 × 0.24 × 0.22	0.21 × 0.17 × 0.05

Data collection				
Diffractometer	Bruker D8 Venture dual source	Bruker D8 Venture dual source	Bruker D8 Venture dual source	Bruker D8 Venture dual source
Absorption correction	Multi-scan (*SADABS*; Krause *et al.*, 2015 ▸)	Multi-scan (*SADABS*; Krause *et al.*, 2015 ▸)	Multi-scan (*SADABS*; Krause *et al.*, 2015 ▸)	Multi-scan (*SADABS*; Krause *et al.*, 2015 ▸)
*T*_min_, *T*_max_	0.890, 0.971	0.939, 0.971	0.931, 0.971	0.914, 0.959
No. of measured, independent and observed [*I* > 2σ(*I*)] reflections	25982, 3518, 3130	34250, 3810, 3567	28796, 3954, 3617	38650, 3882, 3456
*R*_int_	0.036	0.033	0.034	0.034
(sin θ/λ)_max_ (Å^−1^)	0.650	0.650	0.650	0.650

Refinement				
*R*[*F* ^2^ > 2σ(*F* ^2^)], *wR*(*F* ^2^), *S*	0.034, 0.089, 1.05	0.034, 0.098, 1.05	0.029, 0.074, 1.04	0.031, 0.082, 1.04
No. of reflections	3518	3810	3954	3882
No. of parameters	220	260	243	269
H-atom treatment	H atoms treated by a mixture of independent and constrained refinement	H atoms treated by a mixture of independent and constrained refinement	H atoms treated by a mixture of independent and constrained refinement	H atoms treated by a mixture of independent and constrained refinement
Δρ_max_, Δρ_min_ (e Å^−3^)	0.36, −0.23	0.40, −0.21	0.32, −0.23	0.36, −0.22

Computer programs: *APEX3* (Bruker, 2016 ▸), *SHELXT* (Sheldrick, 2015*a* ▸), *SHELXL2019/2* (Sheldrick, 2015*b* ▸), *XP* in *SHELXTL* (Sheldrick, 2008 ▸), and *publCIF* (Westrip, 2010 ▸).

## supplementary crystallographic information

### 4-(4-Nitrophenyl)piperazinium hydrogen succinate (I) Crystal data

**Table:**

C_10_H_14_N_3_O_2_^+^·C_4_H_5_O_4_^−^	*F*(000) = 1376
*M**_r_* = 325.32	*D*_x_ = 1.408 Mg m^−^^3^
Monoclinic, *C*2/*c*	Mo *K*α radiation, λ = 0.71073 Å
*a* = 25.2747 (12) Å	Cell parameters from 9366 reflections
*b* = 8.0434 (4) Å	θ = 2.7–27.5°
*c* = 15.6617 (5) Å	µ = 0.11 mm^−^^1^
β = 105.384 (2)°	*T* = 90 K
*V* = 3069.9 (2) Å^3^	Cut block, pale yellow
*Z* = 8	0.30 × 0.22 × 0.18 mm

### 4-(4-Nitrophenyl)piperazinium hydrogen succinate (I) Data collection

**Table:**

Bruker D8 Venture dual source diffractometer	3518 independent reflections
Radiation source: microsource	3130 reflections with *I* > 2σ(*I*)
Detector resolution: 7.41 pixels mm^-1^	*R*_int_ = 0.036
φ and ω scans	θ_max_ = 27.5°, θ_min_ = 2.7°
Absorption correction: multi-scan (*SADABS*; Krause *et al.*, 2015)	*h* = −32→32
*T*_min_ = 0.890, *T*_max_ = 0.971	*k* = −10→10
25982 measured reflections	*l* = −20→20

### 4-(4-Nitrophenyl)piperazinium hydrogen succinate (I) Refinement

**Table:**

Refinement on *F*^2^	Primary atom site location: structure-invariant direct methods
Least-squares matrix: full	Secondary atom site location: difference Fourier map
*R*[*F*^2^ > 2σ(*F*^2^)] = 0.034	Hydrogen site location: mixed
*wR*(*F*^2^) = 0.089	H atoms treated by a mixture of independent and constrained refinement
*S* = 1.05	*w* = 1/[σ^2^(*F*_o_^2^) + (0.0401*P*)^2^ + 2.5912*P*] where *P* = (*F*_o_^2^ + 2*F*_c_^2^)/3
3518 reflections	(Δ/σ)_max_ = 0.001
220 parameters	Δρ_max_ = 0.36 e Å^−^^3^
0 restraints	Δρ_min_ = −0.23 e Å^−^^3^

### 4-(4-Nitrophenyl)piperazinium hydrogen succinate (I) Special details

**Table:**

Experimental. The crystal was mounted using polyisobutene oil on the tip of a fine glass fibre, which was fastened in a copper mounting pin with electrical solder. It was placed directly into the cold gas stream of a liquid-nitrogen based cryostat (Hope, 1994; Parkin & Hope, 1998). Diffraction data were collected with the crystal at 90K, which is standard practice in this laboratory for the majority of flash-cooled crystals.
Geometry. All esds (except the esd in the dihedral angle between two l.s. planes) are estimated using the full covariance matrix. The cell esds are taken into account individually in the estimation of esds in distances, angles and torsion angles; correlations between esds in cell parameters are only used when they are defined by crystal symmetry. An approximate (isotropic) treatment of cell esds is used for estimating esds involving l.s. planes.
Refinement. Refinement progress was checked using *Platon* (Spek, 2020) and by an *R*-tensor (Parkin, 2000). The final model was further checked with the IUCr utility *checkCIF*.

### 4-(4-Nitrophenyl)piperazinium hydrogen succinate (I) Fractional atomic coordinates and isotropic or equivalent isotropic displacement parameters (Å^2^)

**Table:**

	*x*	*y*	*z*	*U*_iso_*/*U*_eq_	
O1	0.14051 (3)	0.80996 (12)	0.52439 (6)	0.0280 (2)	
O2	0.19261 (4)	0.91415 (12)	0.64508 (5)	0.0259 (2)	
N1	0.41855 (4)	1.00925 (12)	0.33747 (6)	0.01449 (19)	
H1NA	0.4443 (6)	0.9234 (19)	0.3448 (9)	0.024 (4)*	
H1NB	0.4279 (6)	1.087 (2)	0.3023 (10)	0.027 (4)*	
N2	0.32266 (4)	1.14743 (13)	0.37425 (6)	0.0195 (2)	
N3	0.18160 (4)	0.88788 (12)	0.56439 (6)	0.0193 (2)	
C1	0.42042 (4)	1.07833 (14)	0.42694 (7)	0.0162 (2)	
H1A	0.457208	1.125553	0.454157	0.019*	
H1B	0.413555	0.988494	0.465866	0.019*	
C2	0.37707 (4)	1.21296 (14)	0.41778 (7)	0.0184 (2)	
H2A	0.377322	1.255726	0.477154	0.022*	
H2B	0.385724	1.306506	0.382666	0.022*	
C3	0.32172 (5)	1.09046 (16)	0.28539 (7)	0.0221 (3)	
H3A	0.331073	1.184031	0.250990	0.027*	
H3B	0.284357	1.051561	0.254685	0.027*	
C4	0.36260 (4)	0.94930 (14)	0.28984 (7)	0.0185 (2)	
H4A	0.352357	0.853143	0.321552	0.022*	
H4B	0.362220	0.913189	0.229253	0.022*	
C5	0.28969 (4)	1.07550 (14)	0.42138 (7)	0.0170 (2)	
C6	0.24190 (4)	0.98517 (15)	0.37851 (7)	0.0197 (2)	
H6	0.233839	0.966904	0.316459	0.024*	
C7	0.20687 (4)	0.92328 (14)	0.42470 (7)	0.0187 (2)	
H7	0.175211	0.862077	0.394892	0.022*	
C8	0.21826 (4)	0.95117 (14)	0.51539 (7)	0.0168 (2)	
C9	0.26495 (5)	1.03778 (15)	0.55941 (7)	0.0196 (2)	
H9	0.272248	1.056667	0.621320	0.024*	
C10	0.30066 (4)	1.09637 (15)	0.51408 (7)	0.0191 (2)	
H10	0.333230	1.151757	0.545403	0.023*	
O3	0.49072 (3)	0.75060 (9)	0.36438 (6)	0.01950 (18)	
O4	0.56900 (3)	0.86516 (9)	0.35258 (5)	0.01772 (17)	
O5	0.57855 (3)	0.25137 (9)	0.29079 (5)	0.01729 (17)	
O6	0.53144 (3)	0.13451 (9)	0.37722 (5)	0.01695 (17)	
H6O	0.5484 (8)	0.005 (3)	0.3625 (13)	0.065 (6)*	
C11	0.53782 (4)	0.74028 (12)	0.35582 (6)	0.0127 (2)	
C12	0.56315 (4)	0.57314 (12)	0.34704 (7)	0.0145 (2)	
H12A	0.601509	0.572910	0.384282	0.017*	
H12B	0.564189	0.558197	0.284757	0.017*	
C13	0.53315 (4)	0.42686 (12)	0.37343 (7)	0.0142 (2)	
H13A	0.539998	0.426052	0.438666	0.017*	
H13B	0.493229	0.441729	0.347489	0.017*	
C14	0.55019 (4)	0.26118 (12)	0.34399 (7)	0.0124 (2)	

### 4-(4-Nitrophenyl)piperazinium hydrogen succinate (I) Atomic displacement parameters (Å^2^)

**Table:**

	*U*^11^	*U*^22^	*U*^33^	*U*^12^	*U*^13^	*U*^23^
O1	0.0196 (4)	0.0345 (5)	0.0275 (4)	−0.0090 (4)	0.0021 (3)	0.0038 (4)
O2	0.0250 (4)	0.0339 (5)	0.0192 (4)	−0.0051 (4)	0.0063 (3)	0.0036 (3)
N1	0.0143 (4)	0.0131 (4)	0.0171 (4)	0.0014 (3)	0.0060 (3)	0.0020 (3)
N2	0.0142 (4)	0.0278 (5)	0.0177 (4)	0.0021 (4)	0.0061 (3)	−0.0008 (4)
N3	0.0158 (4)	0.0198 (5)	0.0214 (5)	0.0012 (4)	0.0031 (4)	0.0048 (4)
C1	0.0142 (5)	0.0194 (5)	0.0148 (5)	−0.0003 (4)	0.0035 (4)	0.0003 (4)
C2	0.0181 (5)	0.0182 (5)	0.0213 (5)	−0.0007 (4)	0.0091 (4)	−0.0012 (4)
C3	0.0155 (5)	0.0371 (7)	0.0141 (5)	0.0033 (5)	0.0043 (4)	0.0012 (5)
C4	0.0168 (5)	0.0231 (6)	0.0167 (5)	−0.0043 (4)	0.0064 (4)	−0.0038 (4)
C5	0.0142 (5)	0.0185 (5)	0.0192 (5)	0.0056 (4)	0.0058 (4)	0.0006 (4)
C6	0.0174 (5)	0.0240 (6)	0.0175 (5)	0.0024 (4)	0.0044 (4)	−0.0047 (4)
C7	0.0145 (5)	0.0186 (5)	0.0216 (5)	0.0014 (4)	0.0024 (4)	−0.0030 (4)
C8	0.0145 (5)	0.0165 (5)	0.0194 (5)	0.0031 (4)	0.0046 (4)	0.0031 (4)
C9	0.0186 (5)	0.0236 (6)	0.0151 (5)	−0.0009 (4)	0.0020 (4)	0.0024 (4)
C10	0.0149 (5)	0.0232 (6)	0.0176 (5)	−0.0012 (4)	0.0016 (4)	0.0007 (4)
O3	0.0158 (4)	0.0119 (4)	0.0316 (4)	0.0021 (3)	0.0078 (3)	0.0008 (3)
O4	0.0185 (4)	0.0093 (4)	0.0266 (4)	−0.0009 (3)	0.0080 (3)	−0.0010 (3)
O5	0.0229 (4)	0.0123 (4)	0.0193 (4)	−0.0004 (3)	0.0101 (3)	−0.0018 (3)
O6	0.0197 (4)	0.0089 (3)	0.0245 (4)	0.0002 (3)	0.0097 (3)	0.0013 (3)
C11	0.0155 (5)	0.0099 (5)	0.0113 (4)	0.0003 (4)	0.0010 (4)	−0.0004 (4)
C12	0.0148 (5)	0.0094 (5)	0.0192 (5)	0.0006 (4)	0.0044 (4)	−0.0010 (4)
C13	0.0163 (5)	0.0091 (5)	0.0174 (5)	0.0013 (4)	0.0048 (4)	−0.0003 (4)
C14	0.0120 (4)	0.0102 (5)	0.0131 (4)	0.0001 (4)	−0.0001 (4)	0.0000 (4)

### 4-(4-Nitrophenyl)piperazinium hydrogen succinate (I) Geometric parameters (Å, º)

**Table:**

O1—N3	1.2323 (13)	C6—C7	1.3763 (16)
O2—N3	1.2380 (13)	C6—H6	0.9500
N1—C4	1.4930 (13)	C7—C8	1.3906 (15)
N1—C1	1.4964 (14)	C7—H7	0.9500
N1—H1NA	0.934 (15)	C8—C9	1.3861 (16)
N1—H1NB	0.903 (16)	C9—C10	1.3714 (16)
N2—C5	1.3784 (14)	C9—H9	0.9500
N2—C3	1.4594 (14)	C10—H10	0.9500
N2—C2	1.4618 (14)	O3—C11	1.2356 (13)
N3—C8	1.4437 (14)	O4—C11	1.2860 (13)
C1—C2	1.5199 (15)	O5—C14	1.2374 (13)
C1—H1A	0.9900	O6—H6O	1.17 (2)
C1—H1B	0.9900	O6—C14	1.2901 (13)
C2—H2A	0.9900	O6—H6O	1.17 (2)
C2—H2B	0.9900	C11—C12	1.5109 (14)
C3—C4	1.5241 (16)	C12—C13	1.5154 (14)
C3—H3A	0.9900	C12—H12A	0.9900
C3—H3B	0.9900	C12—H12B	0.9900
C4—H4A	0.9900	C13—C14	1.5097 (14)
C4—H4B	0.9900	C13—H13A	0.9900
C5—C10	1.4139 (15)	C13—H13B	0.9900
C5—C6	1.4170 (16)		
			
C4—N1—C1	112.23 (8)	C10—C5—C6	117.24 (10)
C4—N1—H1NA	111.0 (9)	C7—C6—C5	121.52 (10)
C1—N1—H1NA	108.1 (9)	C7—C6—H6	119.2
C4—N1—H1NB	106.6 (9)	C5—C6—H6	119.2
C1—N1—H1NB	111.6 (10)	C6—C7—C8	119.39 (10)
H1NA—N1—H1NB	107.3 (12)	C6—C7—H7	120.3
C5—N2—C3	121.32 (10)	C8—C7—H7	120.3
C5—N2—C2	121.95 (9)	C9—C8—C7	120.48 (10)
C3—N2—C2	109.46 (8)	C9—C8—N3	119.65 (10)
O1—N3—O2	122.46 (10)	C7—C8—N3	119.87 (10)
O1—N3—C8	118.84 (9)	C10—C9—C8	120.37 (10)
O2—N3—C8	118.70 (9)	C10—C9—H9	119.8
N1—C1—C2	109.46 (8)	C8—C9—H9	119.8
N1—C1—H1A	109.8	C9—C10—C5	120.93 (10)
C2—C1—H1A	109.8	C9—C10—H10	119.5
N1—C1—H1B	109.8	C5—C10—H10	119.5
C2—C1—H1B	109.8	H6O—O6—C14	115.4 (10)
H1A—C1—H1B	108.2	H6O—O6—H6O	0 (3)
N2—C2—C1	110.65 (9)	C14—O6—H6O	115.4 (10)
N2—C2—H2A	109.5	O3—C11—O4	124.74 (9)
C1—C2—H2A	109.5	O3—C11—C12	120.89 (9)
N2—C2—H2B	109.5	O4—C11—C12	114.37 (9)
C1—C2—H2B	109.5	C11—C12—C13	114.29 (8)
H2A—C2—H2B	108.1	C11—C12—H12A	108.7
N2—C3—C4	110.55 (9)	C13—C12—H12A	108.7
N2—C3—H3A	109.5	C11—C12—H12B	108.7
C4—C3—H3A	109.5	C13—C12—H12B	108.7
N2—C3—H3B	109.5	H12A—C12—H12B	107.6
C4—C3—H3B	109.5	C14—C13—C12	113.47 (8)
H3A—C3—H3B	108.1	C14—C13—H13A	108.9
N1—C4—C3	108.84 (9)	C12—C13—H13A	108.9
N1—C4—H4A	109.9	C14—C13—H13B	108.9
C3—C4—H4A	109.9	C12—C13—H13B	108.9
N1—C4—H4B	109.9	H13A—C13—H13B	107.7
C3—C4—H4B	109.9	O5—C14—O6	124.17 (9)
H4A—C4—H4B	108.3	O5—C14—C13	121.67 (9)
N2—C5—C10	121.22 (10)	O6—C14—C13	114.14 (9)
N2—C5—C6	121.46 (10)		
			
C4—N1—C1—C2	−54.52 (12)	O1—N3—C8—C9	−179.41 (10)
C5—N2—C2—C1	89.04 (12)	O2—N3—C8—C9	0.67 (16)
C3—N2—C2—C1	−61.41 (12)	O1—N3—C8—C7	−0.13 (15)
N1—C1—C2—N2	57.02 (11)	O2—N3—C8—C7	179.94 (10)
C5—N2—C3—C4	−88.50 (12)	C7—C8—C9—C10	−0.33 (17)
C2—N2—C3—C4	62.17 (12)	N3—C8—C9—C10	178.94 (10)
C1—N1—C4—C3	54.94 (11)	C8—C9—C10—C5	2.44 (18)
N2—C3—C4—N1	−58.37 (12)	N2—C5—C10—C9	173.84 (11)
C3—N2—C5—C10	162.34 (10)	C6—C5—C10—C9	−2.92 (17)
C2—N2—C5—C10	15.32 (16)	O3—C11—C12—C13	−15.24 (14)
C3—N2—C5—C6	−21.04 (16)	O4—C11—C12—C13	165.46 (9)
C2—N2—C5—C6	−168.06 (10)	C11—C12—C13—C14	166.06 (8)
N2—C5—C6—C7	−175.36 (10)	H6O—O6—C14—O5	9.0 (11)
C10—C5—C6—C7	1.39 (16)	H6O—O6—C14—C13	−172.7 (11)
C5—C6—C7—C8	0.62 (17)	C12—C13—C14—O5	−11.74 (14)
C6—C7—C8—C9	−1.19 (17)	C12—C13—C14—O6	169.97 (9)
C6—C7—C8—N3	179.54 (10)		

### 4-(4-Nitrophenyl)piperazinium hydrogen succinate (I) Hydrogen-bond geometry (Å, º)

**Table:**

*D*—H···*A*	*D*—H	H···*A*	*D*···*A*	*D*—H···*A*
N1—H1*NA*···O3	0.934 (15)	1.793 (16)	2.7250 (12)	175.5 (13)
N1—H1*NB*···O5^i^	0.903 (16)	1.945 (16)	2.8127 (12)	160.5 (13)
O6—H6*O*···O4^ii^	1.17 (2)	1.27 (2)	2.4367 (10)	176 (2)
C1—H1*A*···O6^iii^	0.99	2.49	3.1374 (13)	123
C3—H3*A*···O5^i^	0.99	2.59	3.3238 (15)	130
C3—H3*B*···O2^iv^	0.99	2.51	3.4168 (15)	153
C4—H4*A*···O2^v^	0.99	2.55	3.5053 (15)	162
C4—H4*B*···O4^vi^	0.99	2.45	3.2354 (13)	136
C10—H10···O4^vii^	0.95	2.57	3.4146 (13)	149

Symmetry codes: (i) −*x*+1, *y*+1, −*z*+1/2; (ii) *x*, *y*−1, *z*; (iii) *x*, *y*+1, *z*; (iv) *x*, −*y*+2, *z*−1/2; (v) −*x*+1/2, −*y*+3/2, −*z*+1; (vi) −*x*+1, *y*, −*z*+1/2; (vii) −*x*+1, −*y*+2, −*z*+1.

### 4-(4-Nitrophenyl)piperazinium 4-aminobenzoate monohydrate (II) Crystal data

**Table:**

C_10_H_14_N_3_O_2_^+^·C_7_H_6_NO_2_^−^·H_2_O	*Z* = 2
*M**_r_* = 362.38	*F*(000) = 384
Triclinic, *P*1	*D*_x_ = 1.439 Mg m^−^^3^
*a* = 6.0453 (3) Å	Mo *K*α radiation, λ = 0.71073 Å
*b* = 7.3930 (3) Å	Cell parameters from 9362 reflections
*c* = 19.1439 (6) Å	θ = 2.8–27.5°
α = 79.482 (2)°	µ = 0.11 mm^−^^1^
β = 89.215 (1)°	*T* = 90 K
γ = 83.967 (1)°	Cut block, pale yellow
*V* = 836.55 (6) Å^3^	0.30 × 0.26 × 0.25 mm

### 4-(4-Nitrophenyl)piperazinium 4-aminobenzoate monohydrate (II) Data collection

**Table:**

Bruker D8 Venture dual source diffractometer	3810 independent reflections
Radiation source: microsource	3567 reflections with *I* > 2σ(*I*)
Detector resolution: 7.41 pixels mm^-1^	*R*_int_ = 0.033
φ and ω scans	θ_max_ = 27.5°, θ_min_ = 2.2°
Absorption correction: multi-scan (*SADABS*; Krause *et al.*, 2015)	*h* = −7→7
*T*_min_ = 0.939, *T*_max_ = 0.971	*k* = −9→9
34250 measured reflections	*l* = −23→24

### 4-(4-Nitrophenyl)piperazinium 4-aminobenzoate monohydrate (II) Refinement

**Table:**

Refinement on *F*^2^	Secondary atom site location: difference Fourier map
Least-squares matrix: full	Hydrogen site location: mixed
*R*[*F*^2^ > 2σ(*F*^2^)] = 0.034	H atoms treated by a mixture of independent and constrained refinement
*wR*(*F*^2^) = 0.098	*w* = 1/[σ^2^(*F*_o_^2^) + (0.0553*P*)^2^ + 0.2799*P*] where *P* = (*F*_o_^2^ + 2*F*_c_^2^)/3
*S* = 1.05	(Δ/σ)_max_ = 0.001
3810 reflections	Δρ_max_ = 0.40 e Å^−^^3^
260 parameters	Δρ_min_ = −0.21 e Å^−^^3^
0 restraints	Extinction correction: SHELXL-2019/2 (Sheldrick 2015b), Fc^*^=kFc[1+0.001xFc^2^λ^3^/sin(2θ)]^-1/4^
Primary atom site location: structure-invariant direct methods	Extinction coefficient: 0.060 (12)

### 4-(4-Nitrophenyl)piperazinium 4-aminobenzoate monohydrate (II) Special details

**Table:**

Experimental. The crystal was mounted using polyisobutene oil on the tip of a fine glass fibre, which was fastened in a copper mounting pin with electrical solder. It was placed directly into the cold gas stream of a liquid-nitrogen based cryostat (Hope, 1994; Parkin & Hope, 1998). Diffraction data were collected with the crystal at 90K, which is standard practice in this laboratory for the majority of flash-cooled crystals.
Geometry. All esds (except the esd in the dihedral angle between two l.s. planes) are estimated using the full covariance matrix. The cell esds are taken into account individually in the estimation of esds in distances, angles and torsion angles; correlations between esds in cell parameters are only used when they are defined by crystal symmetry. An approximate (isotropic) treatment of cell esds is used for estimating esds involving l.s. planes.
Refinement. Refinement progress was checked using *Platon* (Spek, 2020) and by an *R*-tensor (Parkin, 2000). The final model was further checked with the IUCr utility *checkCIF*.

### 4-(4-Nitrophenyl)piperazinium 4-aminobenzoate monohydrate (II) Fractional atomic coordinates and isotropic or equivalent isotropic displacement parameters (Å^2^)

**Table:**

	*x*	*y*	*z*	*U*_iso_*/*U*_eq_	
O1	0.53605 (15)	0.20260 (13)	1.00635 (4)	0.0313 (2)	
O2	0.23072 (15)	0.38290 (13)	0.99145 (5)	0.0317 (2)	
N1	0.76952 (13)	0.71108 (11)	0.54685 (4)	0.01334 (18)	
H1NA	0.813 (3)	0.774 (2)	0.5028 (9)	0.034 (4)*	
H1NB	0.732 (2)	0.596 (2)	0.5393 (7)	0.023 (3)*	
N2	0.68305 (13)	0.65351 (11)	0.69771 (4)	0.01221 (18)	
N3	0.41469 (16)	0.32803 (12)	0.97077 (5)	0.0193 (2)	
C1	0.57897 (16)	0.81697 (13)	0.57606 (5)	0.0146 (2)	
H1C	0.454845	0.844383	0.541278	0.018*	
H1D	0.624218	0.935914	0.584689	0.018*	
C2	0.50264 (15)	0.70618 (13)	0.64495 (5)	0.0137 (2)	
H2A	0.379688	0.780329	0.664798	0.016*	
H2B	0.444540	0.592986	0.635118	0.016*	
C3	0.88481 (15)	0.56519 (13)	0.66941 (5)	0.0143 (2)	
H3A	0.857762	0.439718	0.662658	0.017*	
H3B	1.007080	0.551797	0.704450	0.017*	
C4	0.95609 (16)	0.67460 (13)	0.59914 (5)	0.0149 (2)	
H4A	1.003928	0.793496	0.606856	0.018*	
H4B	1.084141	0.604189	0.580091	0.018*	
C5	0.61991 (16)	0.57294 (13)	0.76590 (5)	0.01256 (19)	
C6	0.76269 (16)	0.43863 (14)	0.81014 (5)	0.0167 (2)	
H6	0.905917	0.401074	0.793498	0.020*	
C7	0.69753 (17)	0.36046 (14)	0.87753 (5)	0.0182 (2)	
H7	0.795230	0.270086	0.907019	0.022*	
C8	0.48877 (17)	0.41525 (13)	0.90149 (5)	0.0158 (2)	
C9	0.34481 (17)	0.54901 (14)	0.86003 (5)	0.0170 (2)	
H9	0.202792	0.586433	0.877573	0.020*	
C10	0.41010 (16)	0.62734 (13)	0.79291 (5)	0.0159 (2)	
H10	0.311958	0.719530	0.764402	0.019*	
O3	0.91205 (12)	0.87584 (10)	0.41666 (4)	0.01794 (17)	
O4	0.63869 (13)	0.73092 (11)	0.38233 (4)	0.02436 (19)	
N4	0.99454 (17)	1.07521 (13)	0.07778 (5)	0.0216 (2)	
H4NA	0.881 (3)	1.094 (2)	0.0482 (9)	0.039 (4)*	
H4NB	1.098 (3)	1.152 (2)	0.0683 (9)	0.036 (4)*	
C11	0.79522 (16)	0.82924 (13)	0.36896 (5)	0.0147 (2)	
C12	0.84904 (15)	0.89608 (12)	0.29267 (5)	0.0132 (2)	
C13	1.04220 (16)	0.98073 (13)	0.27286 (5)	0.0139 (2)	
H13	1.142098	0.996856	0.308568	0.017*	
C14	1.09049 (16)	1.04151 (13)	0.20203 (5)	0.0155 (2)	
H14	1.223476	1.097509	0.189691	0.019*	
C15	0.94427 (17)	1.02089 (13)	0.14843 (5)	0.0155 (2)	
C16	0.74953 (17)	0.93703 (14)	0.16826 (5)	0.0171 (2)	
H16	0.647635	0.923274	0.132721	0.020*	
C17	0.70465 (16)	0.87434 (13)	0.23895 (5)	0.0154 (2)	
H17	0.573636	0.815474	0.251325	0.019*	
O1W	0.29390 (13)	0.65167 (10)	0.46397 (4)	0.01758 (17)	
H1W1	0.183 (3)	0.730 (2)	0.4450 (9)	0.037 (4)*	
H2W1	0.410 (3)	0.681 (2)	0.4372 (9)	0.041 (4)*	

### 4-(4-Nitrophenyl)piperazinium 4-aminobenzoate monohydrate (II) Atomic displacement parameters (Å^2^)

**Table:**

	*U*^11^	*U*^22^	*U*^33^	*U*^12^	*U*^13^	*U*^23^
O1	0.0317 (5)	0.0350 (5)	0.0201 (4)	0.0004 (4)	0.0010 (3)	0.0114 (3)
O2	0.0324 (5)	0.0330 (5)	0.0247 (4)	0.0021 (4)	0.0150 (3)	0.0036 (3)
N1	0.0154 (4)	0.0138 (4)	0.0104 (4)	−0.0018 (3)	0.0009 (3)	−0.0012 (3)
N2	0.0114 (4)	0.0134 (4)	0.0108 (4)	−0.0001 (3)	0.0004 (3)	−0.0004 (3)
N3	0.0251 (5)	0.0189 (4)	0.0139 (4)	−0.0055 (3)	0.0030 (3)	−0.0014 (3)
C1	0.0158 (4)	0.0143 (4)	0.0125 (4)	0.0010 (3)	0.0001 (3)	−0.0009 (3)
C2	0.0121 (4)	0.0163 (4)	0.0117 (4)	0.0005 (3)	−0.0001 (3)	−0.0008 (3)
C3	0.0124 (4)	0.0168 (4)	0.0122 (4)	0.0009 (3)	0.0014 (3)	−0.0001 (3)
C4	0.0130 (4)	0.0182 (5)	0.0128 (4)	−0.0020 (3)	0.0010 (3)	−0.0009 (3)
C5	0.0148 (4)	0.0114 (4)	0.0118 (4)	−0.0027 (3)	0.0005 (3)	−0.0021 (3)
C6	0.0148 (4)	0.0189 (5)	0.0147 (4)	0.0007 (4)	0.0013 (3)	−0.0001 (4)
C7	0.0195 (5)	0.0190 (5)	0.0141 (4)	0.0002 (4)	−0.0010 (4)	0.0013 (4)
C8	0.0214 (5)	0.0152 (4)	0.0110 (4)	−0.0049 (4)	0.0026 (4)	−0.0014 (3)
C9	0.0176 (5)	0.0160 (5)	0.0169 (5)	−0.0009 (4)	0.0046 (4)	−0.0029 (4)
C10	0.0168 (5)	0.0142 (4)	0.0151 (4)	0.0015 (3)	0.0017 (4)	−0.0003 (3)
O3	0.0217 (4)	0.0183 (4)	0.0128 (3)	−0.0012 (3)	0.0020 (3)	−0.0006 (3)
O4	0.0247 (4)	0.0269 (4)	0.0211 (4)	−0.0106 (3)	0.0074 (3)	0.0006 (3)
N4	0.0260 (5)	0.0244 (5)	0.0130 (4)	−0.0024 (4)	0.0022 (4)	0.0000 (3)
C11	0.0151 (4)	0.0116 (4)	0.0155 (4)	0.0021 (3)	0.0036 (3)	0.0001 (3)
C12	0.0145 (4)	0.0107 (4)	0.0131 (4)	0.0007 (3)	0.0023 (3)	−0.0005 (3)
C13	0.0145 (4)	0.0131 (4)	0.0141 (4)	−0.0010 (3)	0.0005 (3)	−0.0027 (3)
C14	0.0151 (4)	0.0147 (4)	0.0163 (5)	−0.0030 (3)	0.0036 (3)	−0.0017 (3)
C15	0.0196 (5)	0.0125 (4)	0.0134 (4)	0.0013 (3)	0.0025 (3)	−0.0013 (3)
C16	0.0190 (5)	0.0158 (4)	0.0163 (5)	−0.0012 (4)	−0.0031 (4)	−0.0027 (4)
C17	0.0136 (4)	0.0130 (4)	0.0191 (5)	−0.0019 (3)	0.0007 (3)	−0.0012 (3)
O1W	0.0171 (4)	0.0180 (4)	0.0176 (4)	−0.0034 (3)	0.0037 (3)	−0.0023 (3)

### 4-(4-Nitrophenyl)piperazinium 4-aminobenzoate monohydrate (II) Geometric parameters (Å, º)

**Table:**

O1—N3	1.2256 (12)	C7—C8	1.3827 (14)
O2—N3	1.2288 (12)	C7—H7	0.9500
N1—C1	1.4850 (12)	C8—C9	1.3845 (14)
N1—C4	1.4884 (12)	C9—C10	1.3801 (13)
N1—H1NA	0.936 (17)	C9—H9	0.9500
N1—H1NB	0.942 (15)	C10—H10	0.9500
N2—C5	1.3980 (12)	O3—C11	1.2787 (13)
N2—C3	1.4668 (12)	O4—C11	1.2488 (13)
N2—C2	1.4694 (12)	N4—C15	1.3781 (13)
N3—C8	1.4517 (12)	N4—H4NA	0.879 (18)
C1—C2	1.5137 (12)	N4—H4NB	0.886 (18)
C1—H1C	0.9900	C11—C12	1.4968 (12)
C1—H1D	0.9900	C12—C13	1.3978 (13)
C2—H2A	0.9900	C12—C17	1.3999 (13)
C2—H2B	0.9900	C13—C14	1.3856 (13)
C3—C4	1.5197 (12)	C13—H13	0.9500
C3—H3A	0.9900	C14—C15	1.4028 (14)
C3—H3B	0.9900	C14—H14	0.9500
C4—H4A	0.9900	C15—C16	1.4034 (14)
C4—H4B	0.9900	C16—C17	1.3810 (14)
C5—C6	1.4076 (13)	C16—H16	0.9500
C5—C10	1.4102 (13)	C17—H17	0.9500
C6—C7	1.3838 (13)	O1W—H1W1	0.877 (18)
C6—H6	0.9500	O1W—H2W1	0.885 (18)
			
C1—N1—C4	108.92 (7)	C7—C6—H6	119.5
C1—N1—H1NA	111.3 (10)	C5—C6—H6	119.5
C4—N1—H1NA	111.3 (10)	C8—C7—C6	119.32 (9)
C1—N1—H1NB	111.3 (9)	C8—C7—H7	120.3
C4—N1—H1NB	107.5 (8)	C6—C7—H7	120.3
H1NA—N1—H1NB	106.4 (13)	C7—C8—C9	121.45 (9)
C5—N2—C3	116.22 (7)	C7—C8—N3	119.79 (9)
C5—N2—C2	115.77 (7)	C9—C8—N3	118.74 (9)
C3—N2—C2	112.92 (7)	C10—C9—C8	119.20 (9)
O1—N3—O2	122.35 (9)	C10—C9—H9	120.4
O1—N3—C8	119.31 (9)	C8—C9—H9	120.4
O2—N3—C8	118.34 (9)	C9—C10—C5	121.17 (9)
N1—C1—C2	110.02 (7)	C9—C10—H10	119.4
N1—C1—H1C	109.7	C5—C10—H10	119.4
C2—C1—H1C	109.7	C15—N4—H4NA	115.6 (11)
N1—C1—H1D	109.7	C15—N4—H4NB	116.4 (11)
C2—C1—H1D	109.7	H4NA—N4—H4NB	116.6 (15)
H1C—C1—H1D	108.2	O4—C11—O3	123.77 (9)
N2—C2—C1	112.23 (8)	O4—C11—C12	118.01 (9)
N2—C2—H2A	109.2	O3—C11—C12	118.21 (9)
C1—C2—H2A	109.2	C13—C12—C17	118.22 (9)
N2—C2—H2B	109.2	C13—C12—C11	121.69 (9)
C1—C2—H2B	109.2	C17—C12—C11	120.10 (9)
H2A—C2—H2B	107.9	C14—C13—C12	121.14 (9)
N2—C3—C4	112.56 (8)	C14—C13—H13	119.4
N2—C3—H3A	109.1	C12—C13—H13	119.4
C4—C3—H3A	109.1	C13—C14—C15	120.42 (9)
N2—C3—H3B	109.1	C13—C14—H14	119.8
C4—C3—H3B	109.1	C15—C14—H14	119.8
H3A—C3—H3B	107.8	N4—C15—C14	120.82 (9)
N1—C4—C3	110.60 (8)	N4—C15—C16	120.62 (9)
N1—C4—H4A	109.5	C14—C15—C16	118.51 (9)
C3—C4—H4A	109.5	C17—C16—C15	120.61 (9)
N1—C4—H4B	109.5	C17—C16—H16	119.7
C3—C4—H4B	109.5	C15—C16—H16	119.7
H4A—C4—H4B	108.1	C16—C17—C12	121.09 (9)
N2—C5—C6	121.76 (9)	C16—C17—H17	119.5
N2—C5—C10	120.38 (8)	C12—C17—H17	119.5
C6—C5—C10	117.85 (9)	H1W1—O1W—H2W1	104.7 (15)
C7—C6—C5	120.98 (9)		
			
C4—N1—C1—C2	−60.5 (1)	O2—N3—C8—C9	−3.55 (14)
C5—N2—C2—C1	171.62 (8)	C7—C8—C9—C10	0.95 (16)
C3—N2—C2—C1	−50.91 (10)	N3—C8—C9—C10	−177.35 (9)
N1—C1—C2—N2	56.6 (1)	C8—C9—C10—C5	0.26 (15)
C5—N2—C3—C4	−173.16 (8)	N2—C5—C10—C9	179.86 (9)
C2—N2—C3—C4	49.57 (11)	C6—C5—C10—C9	−1.23 (15)
C1—N1—C4—C3	59.29 (10)	O4—C11—C12—C13	−169.47 (9)
N2—C3—C4—N1	−54.06 (10)	O3—C11—C12—C13	10.47 (13)
C3—N2—C5—C6	11.92 (13)	O4—C11—C12—C17	10.42 (14)
C2—N2—C5—C6	147.97 (9)	O3—C11—C12—C17	−169.64 (9)
C3—N2—C5—C10	−169.20 (8)	C17—C12—C13—C14	−0.09 (14)
C2—N2—C5—C10	−33.16 (12)	C11—C12—C13—C14	179.80 (8)
N2—C5—C6—C7	179.93 (9)	C12—C13—C14—C15	0.67 (14)
C10—C5—C6—C7	1.03 (15)	C13—C14—C15—N4	−177.78 (9)
C5—C6—C7—C8	0.12 (16)	C13—C14—C15—C16	−0.24 (14)
C6—C7—C8—C9	−1.14 (16)	N4—C15—C16—C17	176.78 (9)
C6—C7—C8—N3	177.14 (9)	C14—C15—C16—C17	−0.77 (14)
O1—N3—C8—C7	−2.18 (15)	C15—C16—C17—C12	1.38 (15)
O2—N3—C8—C7	178.12 (10)	C13—C12—C17—C16	−0.93 (14)
O1—N3—C8—C9	176.15 (10)	C11—C12—C17—C16	179.18 (8)

### 4-(4-Nitrophenyl)piperazinium 4-aminobenzoate monohydrate (II) Hydrogen-bond geometry (Å, º)

**Table:**

*D*—H···*A*	*D*—H	H···*A*	*D*···*A*	*D*—H···*A*
N1—H1*NA*···O3	0.936 (17)	1.804 (17)	2.737 (1)	174.7 (15)
N1—H1*NB*···O1*W*^i^	0.942 (15)	1.864 (15)	2.7934 (11)	168.4 (13)
N4—H4*NA*···O1^ii^	0.879 (18)	2.258 (18)	3.0861 (13)	156.9 (15)
N4—H4*NB*···O2^iii^	0.886 (18)	2.248 (17)	3.0315 (13)	147.3 (14)
O1*W*—H1*W*1···O3^iv^	0.877 (18)	1.890 (18)	2.7569 (11)	169.7 (16)
O1*W*—H2*W*1···O4	0.885 (18)	1.755 (18)	2.6388 (11)	177.2 (17)
C1—H1*C*···O1*W*	0.99	2.50	3.2511 (12)	132
C2—H2*B*···O4^i^	0.99	2.58	3.5572 (13)	170
C4—H4*A*···O3^v^	0.99	2.51	3.4578 (12)	161
C4—H4*B*···O1*W*^vi^	0.99	2.53	3.2921 (12)	134

Symmetry codes: (i) −*x*+1, −*y*+1, −*z*+1; (ii) *x*, *y*+1, *z*−1; (iii) *x*+1, *y*+1, *z*−1; (iv) *x*−1, *y*, *z*; (v) −*x*+2, −*y*+2, −*z*+1; (vi) *x*+1, *y*, *z*.

### 4-(4-Nitrophenyl)piperazinium 2-(4-chlorophenyl)acetate (III) Crystal data

**Table:**

C_10_H_14_N_3_O_2_^+^·C_8_H_6_ClO_2_^−^	*Z* = 2
*M**_r_* = 377.82	*F*(000) = 396
Triclinic, *P*1	*D*_x_ = 1.451 Mg m^−^^3^
*a* = 6.8051 (2) Å	Mo *K*α radiation, λ = 0.71073 Å
*b* = 9.3927 (5) Å	Cell parameters from 9810 reflections
*c* = 14.3869 (7) Å	θ = 2.6–27.5°
α = 83.849 (2)°	µ = 0.25 mm^−^^1^
β = 81.283 (2)°	*T* = 90 K
γ = 72.492 (2)°	Rounded block, pale yellow
*V* = 865.01 (7) Å^3^	0.28 × 0.24 × 0.22 mm

### 4-(4-Nitrophenyl)piperazinium 2-(4-chlorophenyl)acetate (III) Data collection

**Table:**

Bruker D8 Venture dual source diffractometer	3954 independent reflections
Radiation source: microsource	3617 reflections with *I* > 2σ(*I*)
Detector resolution: 7.41 pixels mm^-1^	*R*_int_ = 0.034
φ and ω scans	θ_max_ = 27.5°, θ_min_ = 2.3°
Absorption correction: multi-scan (*SADABS*; Krause *et al.*, 2015)	*h* = −7→8
*T*_min_ = 0.931, *T*_max_ = 0.971	*k* = −12→12
28796 measured reflections	*l* = −18→18

### 4-(4-Nitrophenyl)piperazinium 2-(4-chlorophenyl)acetate (III) Refinement

**Table:**

Refinement on *F*^2^	Primary atom site location: structure-invariant direct methods
Least-squares matrix: full	Secondary atom site location: difference Fourier map
*R*[*F*^2^ > 2σ(*F*^2^)] = 0.029	Hydrogen site location: mixed
*wR*(*F*^2^) = 0.074	H atoms treated by a mixture of independent and constrained refinement
*S* = 1.04	*w* = 1/[σ^2^(*F*_o_^2^) + (0.0293*P*)^2^ + 0.3972*P*] where *P* = (*F*_o_^2^ + 2*F*_c_^2^)/3
3954 reflections	(Δ/σ)_max_ = 0.001
243 parameters	Δρ_max_ = 0.32 e Å^−^^3^
0 restraints	Δρ_min_ = −0.23 e Å^−^^3^

### 4-(4-Nitrophenyl)piperazinium 2-(4-chlorophenyl)acetate (III) Special details

**Table:**

Experimental. The crystal was mounted using polyisobutene oil on the tip of a fine glass fibre, which was fastened in a copper mounting pin with electrical solder. It was placed directly into the cold gas stream of a liquid-nitrogen based cryostat (Hope, 1994; Parkin & Hope, 1998). Diffraction data were collected with the crystal at 90K, which is standard practice in this laboratory for the majority of flash-cooled crystals.
Geometry. All esds (except the esd in the dihedral angle between two l.s. planes) are estimated using the full covariance matrix. The cell esds are taken into account individually in the estimation of esds in distances, angles and torsion angles; correlations between esds in cell parameters are only used when they are defined by crystal symmetry. An approximate (isotropic) treatment of cell esds is used for estimating esds involving l.s. planes.
Refinement. Refinement progress was checked using *Platon* (Spek, 2020) and by an *R*-tensor (Parkin, 2000). The final model was further checked with the IUCr utility *checkCIF*.

### 4-(4-Nitrophenyl)piperazinium 2-(4-chlorophenyl)acetate (III) Fractional atomic coordinates and isotropic or equivalent isotropic displacement parameters (Å^2^)

**Table:**

	*x*	*y*	*z*	*U*_iso_*/*U*_eq_	
O1	−0.50792 (15)	0.72253 (11)	−0.02713 (7)	0.0316 (2)	
O2	−0.25948 (17)	0.82570 (11)	−0.05317 (7)	0.0356 (2)	
N1	0.26456 (14)	0.13828 (11)	0.41177 (7)	0.01585 (19)	
H1NA	0.325 (2)	0.0464 (18)	0.4359 (10)	0.026 (4)*	
H1NB	0.269 (2)	0.2061 (18)	0.4542 (11)	0.032 (4)*	
N2	0.07010 (14)	0.32686 (10)	0.26302 (6)	0.01586 (19)	
N3	−0.34059 (17)	0.73363 (11)	−0.01097 (7)	0.0227 (2)	
C1	0.38614 (17)	0.16486 (12)	0.32016 (8)	0.0176 (2)	
H1A	0.395730	0.086035	0.277758	0.021*	
H1B	0.528798	0.159661	0.330426	0.021*	
C2	0.28355 (16)	0.31715 (12)	0.27444 (8)	0.0172 (2)	
H2A	0.283868	0.396692	0.314290	0.021*	
H2B	0.362111	0.331786	0.212161	0.021*	
C3	−0.05253 (16)	0.30313 (12)	0.35323 (7)	0.0156 (2)	
H3A	−0.195330	0.309614	0.342459	0.019*	
H3B	−0.061304	0.382276	0.395239	0.019*	
C4	0.04638 (16)	0.15129 (12)	0.39986 (8)	0.0156 (2)	
H4A	−0.033315	0.138579	0.462142	0.019*	
H4B	0.044013	0.071645	0.360567	0.019*	
C5	−0.02922 (16)	0.43171 (12)	0.19619 (7)	0.0151 (2)	
C6	−0.21686 (17)	0.42157 (12)	0.17151 (8)	0.0176 (2)	
H6	−0.273862	0.345143	0.201503	0.021*	
C7	−0.31899 (17)	0.52046 (12)	0.10471 (8)	0.0183 (2)	
H7	−0.446340	0.513477	0.089059	0.022*	
C8	−0.23292 (18)	0.63078 (12)	0.06044 (7)	0.0177 (2)	
C9	−0.04689 (18)	0.64229 (12)	0.08123 (8)	0.0185 (2)	
H9	0.011220	0.716896	0.049151	0.022*	
C10	0.05405 (17)	0.54344 (12)	0.14959 (8)	0.0168 (2)	
H10	0.181000	0.551621	0.164951	0.020*	
Cl1	1.20054 (4)	−0.01622 (3)	0.85294 (2)	0.02223 (8)	
O3	0.31330 (12)	0.34221 (9)	0.51721 (6)	0.01836 (17)	
O4	0.60380 (12)	0.15406 (8)	0.52384 (6)	0.01855 (17)	
C11	0.48457 (16)	0.27847 (11)	0.54780 (7)	0.0141 (2)	
C12	0.54892 (18)	0.36457 (12)	0.61677 (8)	0.0187 (2)	
H12A	0.600482	0.444591	0.580440	0.022*	
H12B	0.424032	0.413504	0.659594	0.022*	
C13	0.71304 (17)	0.27174 (12)	0.67559 (8)	0.0160 (2)	
C14	0.65746 (17)	0.19582 (12)	0.75951 (8)	0.0176 (2)	
H14	0.514929	0.204580	0.779430	0.021*	
C15	0.80586 (18)	0.10768 (12)	0.81460 (8)	0.0179 (2)	
H15	0.765725	0.057433	0.871881	0.022*	
C16	1.01349 (17)	0.09451 (12)	0.78437 (8)	0.0166 (2)	
C17	1.07373 (17)	0.16949 (12)	0.70198 (8)	0.0176 (2)	
H17	1.216498	0.160159	0.682282	0.021*	
C18	0.92272 (17)	0.25871 (12)	0.64834 (8)	0.0171 (2)	
H18	0.963297	0.311542	0.592260	0.021*	

### 4-(4-Nitrophenyl)piperazinium 2-(4-chlorophenyl)acetate (III) Atomic displacement parameters (Å^2^)

**Table:**

	*U*^11^	*U*^22^	*U*^33^	*U*^12^	*U*^13^	*U*^23^
O1	0.0330 (5)	0.0302 (5)	0.0312 (5)	−0.0051 (4)	−0.0172 (4)	0.0068 (4)
O2	0.0498 (6)	0.0257 (5)	0.0320 (5)	−0.0142 (4)	−0.0119 (4)	0.0144 (4)
N1	0.0159 (4)	0.0129 (4)	0.0185 (5)	−0.0027 (4)	−0.0054 (4)	0.0004 (4)
N2	0.0133 (4)	0.0189 (5)	0.0153 (4)	−0.0052 (3)	−0.0029 (3)	0.0026 (3)
N3	0.0318 (6)	0.0165 (5)	0.0170 (5)	−0.0019 (4)	−0.0061 (4)	0.0004 (4)
C1	0.0143 (5)	0.0182 (5)	0.0198 (5)	−0.0033 (4)	−0.0026 (4)	−0.0023 (4)
C2	0.0144 (5)	0.0203 (5)	0.0178 (5)	−0.0062 (4)	−0.0037 (4)	0.0007 (4)
C3	0.0148 (5)	0.0160 (5)	0.0147 (5)	−0.0033 (4)	−0.0019 (4)	0.0012 (4)
C4	0.0162 (5)	0.0141 (5)	0.0175 (5)	−0.0053 (4)	−0.0043 (4)	0.0004 (4)
C5	0.0168 (5)	0.0150 (5)	0.0127 (5)	−0.0033 (4)	−0.0017 (4)	−0.0017 (4)
C6	0.0188 (5)	0.0176 (5)	0.0173 (5)	−0.0073 (4)	−0.0033 (4)	0.0019 (4)
C7	0.0177 (5)	0.0195 (5)	0.0174 (5)	−0.0043 (4)	−0.0041 (4)	−0.0007 (4)
C8	0.0242 (6)	0.0138 (5)	0.0124 (5)	−0.0012 (4)	−0.0031 (4)	−0.0005 (4)
C9	0.0267 (6)	0.0138 (5)	0.0153 (5)	−0.0073 (4)	−0.0002 (4)	−0.0017 (4)
C10	0.0195 (5)	0.0168 (5)	0.0158 (5)	−0.0072 (4)	−0.0021 (4)	−0.0027 (4)
Cl1	0.02364 (15)	0.02014 (14)	0.02045 (14)	−0.00019 (11)	−0.00884 (10)	0.00015 (10)
O3	0.0165 (4)	0.0167 (4)	0.0219 (4)	−0.0031 (3)	−0.0066 (3)	−0.0009 (3)
O4	0.0207 (4)	0.0131 (4)	0.0216 (4)	−0.0023 (3)	−0.0063 (3)	−0.0023 (3)
C11	0.0171 (5)	0.0121 (5)	0.0138 (5)	−0.0064 (4)	−0.0022 (4)	0.0024 (4)
C12	0.0220 (5)	0.0131 (5)	0.0216 (6)	−0.0029 (4)	−0.0082 (4)	−0.0023 (4)
C13	0.0202 (5)	0.0116 (5)	0.0173 (5)	−0.0042 (4)	−0.0055 (4)	−0.0035 (4)
C14	0.0174 (5)	0.0155 (5)	0.0209 (5)	−0.0053 (4)	−0.0024 (4)	−0.0036 (4)
C15	0.0232 (5)	0.0144 (5)	0.0167 (5)	−0.0064 (4)	−0.0025 (4)	−0.0005 (4)
C16	0.0199 (5)	0.0127 (5)	0.0170 (5)	−0.0019 (4)	−0.0066 (4)	−0.0024 (4)
C17	0.0175 (5)	0.0186 (5)	0.0181 (5)	−0.0061 (4)	−0.0026 (4)	−0.0038 (4)
C18	0.0226 (5)	0.0156 (5)	0.0149 (5)	−0.0076 (4)	−0.0032 (4)	−0.0014 (4)

### 4-(4-Nitrophenyl)piperazinium 2-(4-chlorophenyl)acetate (III) Geometric parameters (Å, º)

**Table:**

O1—N3	1.2322 (14)	C7—C8	1.3904 (16)
O2—N3	1.2224 (14)	C7—H7	0.9500
N1—C1	1.4857 (14)	C8—C9	1.3812 (16)
N1—C4	1.4873 (13)	C9—C10	1.3880 (16)
N1—H1NA	0.894 (16)	C9—H9	0.9500
N1—H1NB	0.937 (16)	C10—H10	0.9500
N2—C5	1.3945 (14)	Cl1—C16	1.7434 (11)
N2—C2	1.4610 (13)	O3—C11	1.2609 (13)
N2—C3	1.4670 (13)	O4—C11	1.2545 (13)
N3—C8	1.4544 (14)	C11—C12	1.5319 (15)
C1—C2	1.5175 (15)	C12—C13	1.5055 (15)
C1—H1A	0.9900	C12—H12A	0.9900
C1—H1B	0.9900	C12—H12B	0.9900
C2—H2A	0.9900	C13—C18	1.3930 (16)
C2—H2B	0.9900	C13—C14	1.3959 (16)
C3—C4	1.5137 (14)	C14—C15	1.3901 (15)
C3—H3A	0.9900	C14—H14	0.9500
C3—H3B	0.9900	C15—C16	1.3860 (16)
C4—H4A	0.9900	C15—H15	0.9500
C4—H4B	0.9900	C16—C17	1.3844 (16)
C5—C10	1.4014 (15)	C17—C18	1.3920 (15)
C5—C6	1.4084 (15)	C17—H17	0.9500
C6—C7	1.3755 (15)	C18—H18	0.9500
C6—H6	0.9500		
			
C1—N1—C4	111.22 (8)	C5—C6—H6	119.4
C1—N1—H1NA	108.7 (10)	C6—C7—C8	118.96 (10)
C4—N1—H1NA	109.3 (10)	C6—C7—H7	120.5
C1—N1—H1NB	109.4 (10)	C8—C7—H7	120.5
C4—N1—H1NB	110.8 (10)	C9—C8—C7	121.62 (10)
H1NA—N1—H1NB	107.3 (13)	C9—C8—N3	119.78 (10)
C5—N2—C2	118.82 (9)	C7—C8—N3	118.58 (10)
C5—N2—C3	117.88 (9)	C8—C9—C10	119.14 (10)
C2—N2—C3	112.05 (8)	C8—C9—H9	120.4
O2—N3—O1	123.01 (10)	C10—C9—H9	120.4
O2—N3—C8	118.49 (10)	C9—C10—C5	120.73 (10)
O1—N3—C8	118.5 (1)	C9—C10—H10	119.6
N1—C1—C2	110.44 (9)	C5—C10—H10	119.6
N1—C1—H1A	109.6	O4—C11—O3	124.59 (10)
C2—C1—H1A	109.6	O4—C11—C12	119.40 (9)
N1—C1—H1B	109.6	O3—C11—C12	115.99 (9)
C2—C1—H1B	109.6	C13—C12—C11	115.29 (9)
H1A—C1—H1B	108.1	C13—C12—H12A	108.5
N2—C2—C1	109.47 (9)	C11—C12—H12A	108.5
N2—C2—H2A	109.8	C13—C12—H12B	108.5
C1—C2—H2A	109.8	C11—C12—H12B	108.5
N2—C2—H2B	109.8	H12A—C12—H12B	107.5
C1—C2—H2B	109.8	C18—C13—C14	118.25 (10)
H2A—C2—H2B	108.2	C18—C13—C12	121.49 (10)
N2—C3—C4	110.37 (9)	C14—C13—C12	120.26 (10)
N2—C3—H3A	109.6	C15—C14—C13	121.52 (10)
C4—C3—H3A	109.6	C15—C14—H14	119.2
N2—C3—H3B	109.6	C13—C14—H14	119.2
C4—C3—H3B	109.6	C16—C15—C14	118.74 (10)
H3A—C3—H3B	108.1	C16—C15—H15	120.6
N1—C4—C3	109.74 (9)	C14—C15—H15	120.6
N1—C4—H4A	109.7	C17—C16—C15	121.2 (1)
C3—C4—H4A	109.7	C17—C16—Cl1	119.80 (9)
N1—C4—H4B	109.7	C15—C16—Cl1	119.00 (9)
C3—C4—H4B	109.7	C16—C17—C18	119.24 (10)
H4A—C4—H4B	108.2	C16—C17—H17	120.4
N2—C5—C10	122.74 (10)	C18—C17—H17	120.4
N2—C5—C6	118.81 (10)	C17—C18—C13	121.03 (10)
C10—C5—C6	118.39 (10)	C17—C18—H18	119.5
C7—C6—C5	121.12 (10)	C13—C18—H18	119.5
C7—C6—H6	119.4		
			
C4—N1—C1—C2	56.92 (11)	O1—N3—C8—C7	2.59 (15)
C5—N2—C2—C1	−158.61 (9)	C7—C8—C9—C10	−1.54 (17)
C3—N2—C2—C1	58.37 (11)	N3—C8—C9—C10	179.95 (10)
N1—C1—C2—N2	−56.83 (11)	C8—C9—C10—C5	0.95 (16)
C5—N2—C3—C4	157.89 (9)	N2—C5—C10—C9	177.79 (10)
C2—N2—C3—C4	−58.71 (11)	C6—C5—C10—C9	0.44 (16)
C1—N1—C4—C3	−56.34 (11)	O4—C11—C12—C13	−20.24 (15)
N2—C3—C4—N1	56.40 (11)	O3—C11—C12—C13	161.46 (10)
C2—N2—C5—C10	−10.02 (15)	C11—C12—C13—C18	94.64 (12)
C3—N2—C5—C10	130.87 (11)	C11—C12—C13—C14	−85.23 (13)
C2—N2—C5—C6	167.32 (10)	C18—C13—C14—C15	−0.76 (16)
C3—N2—C5—C6	−51.78 (14)	C12—C13—C14—C15	179.12 (10)
N2—C5—C6—C7	−178.76 (10)	C13—C14—C15—C16	−0.64 (16)
C10—C5—C6—C7	−1.30 (16)	C14—C15—C16—C17	1.28 (16)
C5—C6—C7—C8	0.76 (17)	C14—C15—C16—Cl1	−179.84 (8)
C6—C7—C8—C9	0.69 (17)	C15—C16—C17—C18	−0.50 (16)
C6—C7—C8—N3	179.21 (10)	Cl1—C16—C17—C18	−179.38 (8)
O2—N3—C8—C9	2.19 (16)	C16—C17—C18—C13	−0.95 (16)
O1—N3—C8—C9	−178.86 (10)	C14—C13—C18—C17	1.56 (15)
O2—N3—C8—C7	−176.36 (11)	C12—C13—C18—C17	−178.32 (10)

### 4-(4-Nitrophenyl)piperazinium 2-(4-chlorophenyl)acetate (III) Hydrogen-bond geometry (Å, º)

**Table:**

*D*—H···*A*	*D*—H	H···*A*	*D*···*A*	*D*—H···*A*
N1—H1*NA*···O4^i^	0.894 (16)	1.848 (16)	2.7252 (12)	166.3 (14)
N1—H1*NB*···O3	0.937 (16)	1.765 (17)	2.6903 (12)	169.0 (15)
C4—H4*A*···O4^ii^	0.99	2.46	3.2539 (14)	137
C7—H7···O1^iii^	0.95	2.59	3.2256 (15)	124
C12—H12*A*···O3^iv^	0.99	2.49	3.4710 (14)	173
C15—H15···O2^v^	0.95	2.37	3.1944 (15)	146
C18—H18···O3^vi^	0.95	2.55	3.2644 (15)	132

Symmetry codes: (i) −*x*+1, −*y*, −*z*+1; (ii) *x*−1, *y*, *z*; (iii) −*x*−1, −*y*+1, −*z*; (iv) −*x*+1, −*y*+1, −*z*+1; (v) *x*+1, *y*−1, *z*+1; (vi) *x*+1, *y*, *z*.

### 4-(4-Nitrophenyl)piperazinium 2,3,4,5,6-pentafluorobenzoate (IV) Crystal data

**Table:**

C_10_H_14_N_3_O_2_^+^·C_7_F_5_O_2_^−^	*Z* = 2
*M**_r_* = 419.31	*F*(000) = 428
Triclinic, *P*1	*D*_x_ = 1.649 Mg m^−^^3^
*a* = 5.9779 (3) Å	Mo *K*α radiation, λ = 0.71073 Å
*b* = 11.3934 (8) Å	Cell parameters from 9830 reflections
*c* = 12.9312 (9) Å	θ = 2.8–27.5°
α = 75.754 (2)°	µ = 0.15 mm^−^^1^
β = 81.670 (2)°	*T* = 90 K
γ = 87.717 (2)°	Tablet, pale yellow
*V* = 844.63 (9) Å^3^	0.21 × 0.17 × 0.05 mm

### 4-(4-Nitrophenyl)piperazinium 2,3,4,5,6-pentafluorobenzoate (IV) Data collection

**Table:**

Bruker D8 Venture dual source diffractometer	3882 independent reflections
Radiation source: microsource	3456 reflections with *I* > 2σ(*I*)
Detector resolution: 7.41 pixels mm^-1^	*R*_int_ = 0.034
φ and ω scans	θ_max_ = 27.5°, θ_min_ = 2.2°
Absorption correction: multi-scan (*SADABS*; Krause *et al.*, 2015)	*h* = −7→7
*T*_min_ = 0.914, *T*_max_ = 0.959	*k* = −14→14
38650 measured reflections	*l* = −16→16

### 4-(4-Nitrophenyl)piperazinium 2,3,4,5,6-pentafluorobenzoate (IV) Refinement

**Table:**

Refinement on *F*^2^	Secondary atom site location: difference Fourier map
Least-squares matrix: full	Hydrogen site location: mixed
*R*[*F*^2^ > 2σ(*F*^2^)] = 0.031	H atoms treated by a mixture of independent and constrained refinement
*wR*(*F*^2^) = 0.082	*w* = 1/[σ^2^(*F*_o_^2^) + (0.0359*P*)^2^ + 0.419*P*] where *P* = (*F*_o_^2^ + 2*F*_c_^2^)/3
*S* = 1.04	(Δ/σ)_max_ < 0.001
3882 reflections	Δρ_max_ = 0.36 e Å^−^^3^
269 parameters	Δρ_min_ = −0.22 e Å^−^^3^
0 restraints	Extinction correction: SHELXL-2019/2 (Sheldrick 2015b), Fc^*^=kFc[1+0.001xFc^2^λ^3^/sin(2θ)]^-1/4^
Primary atom site location: structure-invariant direct methods	Extinction coefficient: 0.008 (2)

### 4-(4-Nitrophenyl)piperazinium 2,3,4,5,6-pentafluorobenzoate (IV) Special details

**Table:**

Experimental. The crystal was mounted using polyisobutene oil on the tip of a fine glass fibre, which was fastened in a copper mounting pin with electrical solder. It was placed directly into the cold gas stream of a liquid-nitrogen based cryostat (Hope, 1994; Parkin & Hope, 1998). Diffraction data were collected with the crystal at 90K, which is standard practice in this laboratory for the majority of flash-cooled crystals.
Geometry. All esds (except the esd in the dihedral angle between two l.s. planes) are estimated using the full covariance matrix. The cell esds are taken into account individually in the estimation of esds in distances, angles and torsion angles; correlations between esds in cell parameters are only used when they are defined by crystal symmetry. An approximate (isotropic) treatment of cell esds is used for estimating esds involving l.s. planes.
Refinement. Refinement progress was checked using *Platon* (Spek, 2020) and by an *R*-tensor (Parkin, 2000). The final model was further checked with the IUCr utility *checkCIF*.

### 4-(4-Nitrophenyl)piperazinium 2,3,4,5,6-pentafluorobenzoate (IV) Fractional atomic coordinates and isotropic or equivalent isotropic displacement parameters (Å^2^)

**Table:**

	*x*	*y*	*z*	*U*_iso_*/*U*_eq_	
O1	0.43180 (17)	0.17458 (10)	−0.07590 (8)	0.0322 (2)	
O2	0.41598 (17)	0.36577 (10)	−0.07776 (8)	0.0315 (2)	
N1	1.18846 (17)	0.19065 (9)	0.40887 (8)	0.0156 (2)	
H1NA	1.080 (3)	0.1911 (13)	0.4679 (12)	0.019*	
H1NB	1.331 (3)	0.1841 (13)	0.4282 (12)	0.019*	
N2	1.23887 (16)	0.20731 (9)	0.18180 (8)	0.0164 (2)	
N3	0.49963 (18)	0.26345 (11)	−0.05166 (8)	0.0241 (2)	
C1	1.16518 (19)	0.30898 (10)	0.32993 (9)	0.0170 (2)	
H1C	1.215721	0.375464	0.357861	0.020*	
H1D	1.004664	0.323501	0.319507	0.020*	
C2	1.30775 (19)	0.30694 (11)	0.22310 (9)	0.0178 (2)	
H2A	1.290093	0.384788	0.170238	0.021*	
H2B	1.469162	0.297174	0.233008	0.021*	
C3	1.2830 (2)	0.09221 (11)	0.25697 (9)	0.0178 (2)	
H3A	1.445777	0.085880	0.264569	0.021*	
H3B	1.244024	0.023963	0.228146	0.021*	
C4	1.1436 (2)	0.08444 (10)	0.36671 (9)	0.0175 (2)	
H4A	0.980836	0.082092	0.360358	0.021*	
H4B	1.181938	0.008818	0.417856	0.021*	
C5	1.04949 (19)	0.22037 (11)	0.12889 (9)	0.0153 (2)	
C6	0.9588 (2)	0.12034 (11)	0.10325 (9)	0.0182 (2)	
H6	1.021849	0.042104	0.126884	0.022*	
C7	0.7802 (2)	0.13394 (12)	0.04448 (10)	0.0198 (2)	
H7	0.721386	0.065866	0.027385	0.024*	
C8	0.68749 (19)	0.24811 (12)	0.01068 (9)	0.0189 (2)	
C9	0.7691 (2)	0.34801 (11)	0.03565 (9)	0.0188 (2)	
H9	0.702687	0.425476	0.012554	0.023*	
C10	0.9478 (2)	0.33452 (11)	0.09439 (9)	0.0174 (2)	
H10	1.003224	0.403213	0.111854	0.021*	
O3	0.86075 (13)	0.21817 (8)	0.56526 (7)	0.01998 (19)	
O4	0.61190 (14)	0.17431 (8)	0.46723 (7)	0.01912 (19)	
C11	0.66662 (18)	0.21484 (10)	0.54141 (9)	0.0144 (2)	
C12	0.48004 (18)	0.26637 (10)	0.61162 (9)	0.0142 (2)	
C13	0.29057 (19)	0.2006 (1)	0.66651 (9)	0.0144 (2)	
C14	0.12144 (19)	0.2486 (1)	0.72922 (9)	0.0152 (2)	
C15	0.13793 (19)	0.36674 (11)	0.73547 (9)	0.0164 (2)	
C16	0.3259 (2)	0.43432 (10)	0.68299 (10)	0.0171 (2)	
C17	0.49472 (19)	0.38314 (10)	0.62342 (9)	0.0161 (2)	
F1	0.26874 (12)	0.08537 (6)	0.66068 (6)	0.01959 (16)	
F2	−0.05531 (12)	0.18192 (6)	0.78430 (6)	0.02050 (16)	
F3	−0.02700 (12)	0.41607 (7)	0.79314 (6)	0.02259 (17)	
F4	0.34328 (13)	0.54896 (7)	0.69035 (6)	0.02478 (18)	
F5	0.67401 (12)	0.45273 (6)	0.57274 (6)	0.02176 (17)	

### 4-(4-Nitrophenyl)piperazinium 2,3,4,5,6-pentafluorobenzoate (IV) Atomic displacement parameters (Å^2^)

**Table:**

	*U*^11^	*U*^22^	*U*^33^	*U*^12^	*U*^13^	*U*^23^
O1	0.0272 (5)	0.0461 (6)	0.0283 (5)	−0.0045 (4)	−0.0106 (4)	−0.0137 (4)
O2	0.0268 (5)	0.0417 (6)	0.0257 (5)	0.0087 (4)	−0.0111 (4)	−0.0048 (4)
N1	0.0123 (4)	0.0197 (5)	0.0159 (5)	0.0007 (4)	−0.0026 (4)	−0.0061 (4)
N2	0.0159 (5)	0.0176 (5)	0.0165 (5)	−0.0003 (4)	−0.0035 (4)	−0.0052 (4)
N3	0.0183 (5)	0.0382 (7)	0.0158 (5)	0.0003 (4)	−0.0032 (4)	−0.0061 (5)
C1	0.0166 (5)	0.0159 (5)	0.0201 (6)	−0.0007 (4)	−0.0034 (4)	−0.0066 (4)
C2	0.0161 (5)	0.0190 (6)	0.0187 (6)	−0.0038 (4)	−0.0026 (4)	−0.0049 (4)
C3	0.0182 (5)	0.0184 (6)	0.0186 (6)	0.0051 (4)	−0.0053 (4)	−0.0070 (4)
C4	0.0182 (5)	0.0156 (5)	0.0189 (6)	0.0005 (4)	−0.0041 (4)	−0.0040 (4)
C5	0.0140 (5)	0.0193 (6)	0.0121 (5)	−0.0005 (4)	0.0003 (4)	−0.0041 (4)
C6	0.0188 (6)	0.0178 (6)	0.0181 (5)	0.0002 (4)	−0.0020 (4)	−0.0050 (4)
C7	0.0193 (6)	0.0242 (6)	0.0172 (5)	−0.0033 (5)	−0.0012 (4)	−0.0078 (5)
C8	0.0144 (5)	0.0292 (6)	0.0130 (5)	−0.0004 (5)	−0.0024 (4)	−0.0044 (5)
C9	0.0175 (5)	0.0213 (6)	0.0152 (5)	0.0020 (4)	−0.0003 (4)	−0.0015 (4)
C10	0.0177 (5)	0.0179 (6)	0.0164 (5)	−0.0012 (4)	−0.0009 (4)	−0.0045 (4)
O3	0.0124 (4)	0.0296 (5)	0.0211 (4)	0.0009 (3)	−0.0036 (3)	−0.0115 (4)
O4	0.0153 (4)	0.0250 (4)	0.0208 (4)	0.0024 (3)	−0.0047 (3)	−0.0118 (3)
C11	0.0136 (5)	0.0136 (5)	0.0159 (5)	0.0003 (4)	−0.0021 (4)	−0.0032 (4)
C12	0.0131 (5)	0.0164 (5)	0.0141 (5)	0.0010 (4)	−0.0041 (4)	−0.0043 (4)
C13	0.0154 (5)	0.0130 (5)	0.0160 (5)	0.0000 (4)	−0.0051 (4)	−0.0040 (4)
C14	0.0134 (5)	0.0180 (6)	0.0133 (5)	−0.0025 (4)	−0.0017 (4)	−0.0019 (4)
C15	0.0152 (5)	0.0201 (6)	0.0149 (5)	0.0035 (4)	−0.0017 (4)	−0.0069 (4)
C16	0.0192 (6)	0.0138 (5)	0.0205 (6)	0.0002 (4)	−0.0043 (4)	−0.0073 (4)
C17	0.0134 (5)	0.0172 (5)	0.0176 (5)	−0.0032 (4)	−0.0017 (4)	−0.0037 (4)
F1	0.0192 (3)	0.0132 (3)	0.0269 (4)	−0.0020 (3)	−0.0013 (3)	−0.0067 (3)
F2	0.0171 (3)	0.0219 (4)	0.0199 (4)	−0.0052 (3)	0.0037 (3)	−0.0033 (3)
F3	0.0201 (4)	0.0244 (4)	0.0240 (4)	0.0027 (3)	0.0036 (3)	−0.0115 (3)
F4	0.0244 (4)	0.0158 (4)	0.0366 (4)	−0.0012 (3)	−0.0006 (3)	−0.0131 (3)
F5	0.0171 (3)	0.0186 (4)	0.0291 (4)	−0.0062 (3)	0.0024 (3)	−0.0071 (3)

### 4-(4-Nitrophenyl)piperazinium 2,3,4,5,6-pentafluorobenzoate (IV) Geometric parameters (Å, º)

**Table:**

O1—N3	1.2304 (15)	C6—C7	1.3806 (17)
O2—N3	1.2376 (15)	C6—H6	0.9500
N1—C4	1.4922 (15)	C7—C8	1.3866 (18)
N1—C1	1.4926 (15)	C7—H7	0.9500
N1—H1NA	0.929 (15)	C8—C9	1.3814 (18)
N1—H1NB	0.915 (16)	C9—C10	1.3810 (17)
N2—C5	1.3902 (14)	C9—H9	0.9500
N2—C2	1.4638 (14)	C10—H10	0.9500
N2—C3	1.4675 (15)	O3—C11	1.2475 (14)
N3—C8	1.4556 (15)	O4—C11	1.2496 (14)
C1—C2	1.5193 (16)	C11—C12	1.5263 (15)
C1—H1C	0.9900	C12—C17	1.3836 (16)
C1—H1D	0.9900	C12—C13	1.3860 (16)
C2—H2A	0.9900	C13—F1	1.3459 (13)
C2—H2B	0.9900	C13—C14	1.3841 (16)
C3—C4	1.5225 (16)	C14—F2	1.3343 (13)
C3—H3A	0.9900	C14—C15	1.3764 (16)
C3—H3B	0.9900	C15—F3	1.3391 (13)
C4—H4A	0.9900	C15—C16	1.3807 (17)
C4—H4B	0.9900	C16—F4	1.3418 (13)
C5—C10	1.4106 (16)	C16—C17	1.3788 (16)
C5—C6	1.4107 (16)	C17—F5	1.3473 (13)
			
C4—N1—C1	113.04 (9)	C10—C5—C6	117.61 (10)
C4—N1—H1NA	107.8 (9)	C7—C6—C5	121.27 (11)
C1—N1—H1NA	106.1 (9)	C7—C6—H6	119.4
C4—N1—H1NB	109.5 (9)	C5—C6—H6	119.4
C1—N1—H1NB	109.6 (9)	C6—C7—C8	119.25 (11)
H1NA—N1—H1NB	110.9 (13)	C6—C7—H7	120.4
C5—N2—C2	119.8 (1)	C8—C7—H7	120.4
C5—N2—C3	120.24 (10)	C9—C8—C7	121.24 (11)
C2—N2—C3	108.81 (9)	C9—C8—N3	119.10 (11)
O1—N3—O2	123.20 (11)	C7—C8—N3	119.66 (11)
O1—N3—C8	118.67 (11)	C10—C9—C8	119.56 (11)
O2—N3—C8	118.13 (11)	C10—C9—H9	120.2
N1—C1—C2	109.46 (9)	C8—C9—H9	120.2
N1—C1—H1C	109.8	C9—C10—C5	121.06 (11)
C2—C1—H1C	109.8	C9—C10—H10	119.5
N1—C1—H1D	109.8	C5—C10—H10	119.5
C2—C1—H1D	109.8	O3—C11—O4	126.84 (11)
H1C—C1—H1D	108.2	O3—C11—C12	115.21 (10)
N2—C2—C1	110.54 (9)	O4—C11—C12	117.95 (10)
N2—C2—H2A	109.5	C17—C12—C13	116.73 (10)
C1—C2—H2A	109.5	C17—C12—C11	120.47 (10)
N2—C2—H2B	109.5	C13—C12—C11	122.8 (1)
C1—C2—H2B	109.5	F1—C13—C14	117.97 (10)
H2A—C2—H2B	108.1	F1—C13—C12	119.72 (10)
N2—C3—C4	110.32 (9)	C14—C13—C12	122.31 (10)
N2—C3—H3A	109.6	F2—C14—C15	119.71 (10)
C4—C3—H3A	109.6	F2—C14—C13	121.1 (1)
N2—C3—H3B	109.6	C15—C14—C13	119.19 (10)
C4—C3—H3B	109.6	F3—C15—C14	120.17 (10)
H3A—C3—H3B	108.1	F3—C15—C16	119.85 (10)
N1—C4—C3	110.66 (9)	C14—C15—C16	119.98 (10)
N1—C4—H4A	109.5	F4—C16—C17	120.49 (10)
C3—C4—H4A	109.5	F4—C16—C15	119.94 (10)
N1—C4—H4B	109.5	C17—C16—C15	119.57 (11)
C3—C4—H4B	109.5	F5—C17—C16	117.56 (10)
H4A—C4—H4B	108.1	F5—C17—C12	120.25 (10)
N2—C5—C10	121.43 (10)	C16—C17—C12	122.14 (11)
N2—C5—C6	120.9 (1)		
			
C4—N1—C1—C2	−52.11 (12)	O4—C11—C12—C17	−124.65 (12)
C5—N2—C2—C1	80.27 (13)	O3—C11—C12—C13	−124.39 (12)
C3—N2—C2—C1	−63.65 (12)	O4—C11—C12—C13	55.62 (15)
N1—C1—C2—N2	58.10 (12)	C17—C12—C13—F1	−178.3 (1)
C5—N2—C3—C4	−81.76 (12)	C11—C12—C13—F1	1.44 (16)
C2—N2—C3—C4	61.97 (12)	C17—C12—C13—C14	0.83 (16)
C1—N1—C4—C3	51.34 (12)	C11—C12—C13—C14	−179.43 (10)
N2—C3—C4—N1	−55.67 (12)	F1—C13—C14—F2	1.56 (16)
C2—N2—C5—C10	13.05 (16)	C12—C13—C14—F2	−177.59 (10)
C3—N2—C5—C10	152.87 (10)	F1—C13—C14—C15	−179.14 (10)
C2—N2—C5—C6	−170.03 (10)	C12—C13—C14—C15	1.72 (17)
C3—N2—C5—C6	−30.21 (15)	F2—C14—C15—F3	−2.64 (16)
N2—C5—C6—C7	−175.64 (11)	C13—C14—C15—F3	178.05 (10)
C10—C5—C6—C7	1.39 (17)	F2—C14—C15—C16	176.77 (10)
C5—C6—C7—C8	−0.41 (18)	C13—C14—C15—C16	−2.54 (17)
C6—C7—C8—C9	−0.66 (18)	F3—C15—C16—F4	0.33 (17)
C6—C7—C8—N3	179.78 (10)	C14—C15—C16—F4	−179.08 (10)
O1—N3—C8—C9	178.16 (11)	F3—C15—C16—C17	−179.76 (10)
O2—N3—C8—C9	−1.79 (16)	C14—C15—C16—C17	0.83 (17)
O1—N3—C8—C7	−2.27 (17)	F4—C16—C17—F5	−0.70 (17)
O2—N3—C8—C7	177.78 (11)	C15—C16—C17—F5	179.4 (1)
C7—C8—C9—C10	0.68 (17)	F4—C16—C17—C12	−178.25 (10)
N3—C8—C9—C10	−179.75 (10)	C15—C16—C17—C12	1.85 (18)
C8—C9—C10—C5	0.35 (17)	C13—C12—C17—F5	179.89 (10)
N2—C5—C10—C9	175.65 (10)	C11—C12—C17—F5	0.15 (16)
C6—C5—C10—C9	−1.36 (17)	C13—C12—C17—C16	−2.62 (17)
O3—C11—C12—C17	55.33 (15)	C11—C12—C17—C16	177.63 (10)

### 4-(4-Nitrophenyl)piperazinium 2,3,4,5,6-pentafluorobenzoate (IV) Hydrogen-bond geometry (Å, º)

**Table:**

*D*—H···*A*	*D*—H	H···*A*	*D*···*A*	*D*—H···*A*
N1—H1*NA*···O3	0.929 (15)	1.754 (16)	2.6723 (13)	169.4 (14)
N1—H1*NB*···O4^i^	0.915 (16)	1.816 (16)	2.7310 (13)	178.8 (14)
C1—H1*C*···F5^ii^	0.99	2.49	3.4813 (14)	177
C1—H1*D*···F4^iii^	0.99	2.49	3.3996 (14)	153
C4—H4*B*···O3^iv^	0.99	2.56	3.3421 (15)	136
C6—H6···F2^v^	0.95	2.54	3.4536 (15)	161

Symmetry codes: (i) *x*+1, *y*, *z*; (ii) −*x*+2, −*y*+1, −*z*+1; (iii) −*x*+1, −*y*+1, −*z*+1; (iv) −*x*+2, −*y*, −*z*+1; (v) −*x*+1, −*y*, −*z*+1.
